# Filling the gap in CNS drug development: evaluation of the role of drug repurposing

**DOI:** 10.1080/20016689.2017.1299833

**Published:** 2017-04-10

**Authors:** A. Caban, K. Pisarczyk, K. Kopacz, A. Kapuśniak, M. Toumi, C. Rémuzat, A. Kornfeld

**Affiliations:** ^a^ Creativ-Ceutical, Pricing & Market Access Department, Krakow, Poland; ^b^ Faculté de Médecine, Laboratoire de Santé Publique, Aix-Marseille Université, Université de la Méditerranée, Marseille Cedex, France; ^c^ Creativ-Ceutical, Pricing & Market Access Department, Paris, France

**Keywords:** Drug repurposing, drug repositioning, drug reprofiling, drug retasking, central nervous system, psychiatry, neurology

## Abstract

**Background and objective**: Background and objective: Drug repurposing has been considered a cost-effective and reduced-risk strategy for developing new drugs. Little is known and documented regarding the efficiency of repurposing strategies in drug development. The objective of this article is to assess the extent and meaning of this process in the CNS area.

**Methods**: In order to identify repurposed drugs that target the CNS, an extensive search was performed. For each identified case, its initial and target indication, development status and the type of repurposing strategy (repositioning, reformulation or both) was recorded.

**Results**: One hundred and eighteen source products were identified. They were repurposed (mainly repositioned) 203 times with 81 products repurposed once and 38 products repurposed twice or more. The highest number of source drugs originated from the CNS area. Alzheimer’s disease was targeted most often. Half of the new indications were approved. Regarding repurposing within the CNS area, epilepsy, schizophrenia and depression were the richest sources of repurposed drugs.

**Conclusions**: Repurposing drugs into CNS is an efficient and very active drug development method, exemplified by the considerable number of new indications that have been found via this strategy, with approximately half of the target indications currently under development.

## Background and objective

Drug repurposing pertains to the development of new therapeutic uses or new formulations for a known drug, or the combination of two or more known drugs that were previously used separately. These drug development strategies are known as drug repositioning, drug reformulation and drug combination, respectively. Within drug repurposing, the two most frequent strategies are drug repositioning and drug reformulation.[1]

Traditionally, drug repurposing has been considered a cost-effective and reduced-risk strategy for developing new drugs, and extending the patent protection period for existing products.[2,3] However, the focus of drug repurposing has been changing in the latest decade. This was largely driven by a change in the regulatory framework applicable to these development strategies, and creation of information-sharing platforms for potential target molecules.[4,5] Several initiatives have been established, allowing information sharing and creation of repurposing partnerships between academia and private companies.[4,5] Increasingly, drug repurposing is seen as a method for rediscovering value in ‘old molecules’ and finding new therapeutic uses, particularly in poorly addressed therapeutic areas, such as oncology, central nervous system (CNS), paediatric or orphan diseases.

CNS seems to be a critical area for drug repurposing.[1] The underlying pathophysiology of CNS disorders is continuously being researched and thus better understood; therefore, it becomes possible to revisit the receptor profiling and mode of action of already commercialised drugs. Indeed, most of the disorders within the CNS, being of psychiatric or neurological kind, are still poorly understood in terms of their underlying pathophysiology and biological mechanisms.[6] To date, the exact mode of action by which many of the currently approved CNS drugs exert their effect is still not fully understood. As such, the current disease characterisation within CNS is very much founded on clinical aspects, rather than the underlying pathophysiology.[7] It is thus unsurprising that, despite the high prevalence of CNS disorders in the overall population and the high unmet need in this area, the discovery and development of drugs for CNS diseases has one of the lowest success rates.[8,9] Several studies concluded that not only is the number of drugs available for clinical development in CNS lower compared with other areas, but also the regulatory approval times are longer.[10–12] In addition, the health technology assessments (HTA) and pricing and reimbursement evaluations, conducted to determine market access conditions for registered drugs, also contribute to delayed availability of these medicines to patients and, in some situations, only partial or no access to them.[13–15]

Although it can be stated that drug repurposing has become a common drug development strategy in the last decade [16] – corresponding to about 30–40% of medicines approved in the USA in 2007–2009 – it is only seldom described in the literature.[1] Currently, little is known and documented regarding drug repurposing in the treatment of CNS diseases and the impact of repurposing in this area remains unknown. Well-known examples of repositioning in the CNS area are amantadine (repositioned from treatment of influenza to treatment of Parkinson disease in the late 1960s) and propranolol (initially approved for treatment of angina and hypertension and repurposed for prophylaxis of migraine in the early 1980s).[17,18] More recently, dimethyl fumarate (sold under the trade name Tecfidera®), considered the most promising orally administered disease modifying product for multiple sclerosis, is a well-known example of drug repositioning from psoriasis.[19]

As such, it is of relevance to evaluate what exact role drug repurposing has played for the already approved CNS drugs, and to understand what potential it can have for future development in this area.

The objective of this article was to quantitatively assess the extent of repurposing within the CNS area. We aimed to identify drugs that underwent, or are undergoing, the repurposing process, and to find their initial and target indication, as well as their development status.

## Methods

In order to identify and gather information on repurposed drugs that target the CNS, a comprehensive literature review was performed. Various complementary sources were searched up to January 2016.

A comprehensive literature search through MEDLINE (In-Process & Other Non-Indexed Citations, Ovid) and EMBASE databases was conducted. The detailed search strategy is described in the Supplementary Material. The retrieved titles and abstracts were screened. Abstracts that appeared to report on particular cases of drug repurposing were included for further full text analysis. References of each publication were checked for additional sources.

The websites of pharmaceutical companies involved in repurposing/reformulation of drugs, as well as the websites of all initiatives undertaken to promote and develop repurposing/reformulation, were reviewed (the ‘Discovering new therapeutic uses for existing molecules’ initiative by National Center for Advancing Translational Sciences at the National Institutes of Health [NIH]; the Rare Disease Repurposing Database by the US Food and Drug Administration’s [FDA] Office of Orphan Products Development; the Indications Discovery & Drug Repositioning Summit; the Annual Drug Repositioning & Indications Discovery Conference; and the World Drug Repositioning Congress).

In addition, the following databases were screened: MedTrack, Google, websites of regulatory agencies (European Medicines Agency [EMA] and the FDA), ClinicalTrials.gov. These databases were used to identify new cases of repurposing, as well as to obtain missing information, for example initial or target indication of previously identified cases. Depending on the information missing, key words included initial or target indication or formulation, followed by the name of the repurposed drug.

Publications and other sources that reported on any cases of drugs repurposed to the CNS area were included. No restrictions on type, date or geographical scope of the publications were applied. Publications that reported on cases which failed in development or had only preclinical (*in vivo, in vitro*, animal) data were excluded.

The repurposing strategies considered included repositioning, defined as ‘a new indication for a known drug’, and reformulation, defined as ‘a new formulation for an existing product’.[1] For combinations of drugs, each individual drug was treated as a separate case with different initial indication. If a drug was repurposed several times, each process was recorded separately and counted as a separate case. Both approved repurposed drugs, as well as candidates for repurposing (in development) were considered appropriate for inclusion. Regarding drugs in development, only the drugs that had already initiated clinical studies were included in the analyses. We have excluded minor reformulations such as for example tablets to prolonged release tablets, or syrup to drops, as well as new strengths. Only major reformulations that lead to different route of administration (e.g. oral to intramuscular or oral to transdermal) were taken into account.

Cases that met the inclusion criteria were extracted from the source material and placed into a descriptive table in alphabetical order. For each case, initial therapeutic area, and initial and target indication were recorded. Each case was classified by development status (approved or in development), as well as by the type of repurposing strategy (reformulation, repositioning or both).

Cases were analysed according to their development status (approved vs. in development), type of repurposing (reformulation vs. repositioning vs. both) as well as the number of repurposing episodes (one vs. two vs. three or more). In addition, cases were grouped according to initial therapeutic area and target therapeutic indication. Separate analysis using initial therapeutic indications (more detailed than initial therapeutic area) was performed for drugs that were repurposed within the CNS.

## Results

In total, 172 information sources were included in the analysis. There were 65 publications, 39 ClinicalTrials.gov links, 30 Medtrack Reports, 24 regulatory and medical research agency materials (from the EMA, FDA, NIH, and the UK Medicines and Healthcare products Regulatory Agency [MHRA], 6 manufacturers’ websites and 8 other sources. The list of included sources can be found in the Supplementary Material.

This study has identified 118 source products. They were repurposed 203 times (203 cases) with 80 products repurposed once, 16 products repurposed twice and 22 products repurposed three times or more. The details of all identified cases are listed in the Supplementary Material. Among products repurposed multiple times, over two thirds (68%) came from the CNS area. Half of the new indications (102 cases) were approved. The majority of approved cases (80%) originated from the CNS area, while the majority (61%) of cases in development originated from areas outside the CNS. Most of the cases were repositioned (171), while only 16 were reformulated, and 16 were reformulated and repositioned at the same time. All results are presented in Table 1.

**Table 1.** The number of source products and target indications included in the analysis.

**Source products**	**118**
Products repurposed once	80
Products repurposed twice	16
Products repurposed three times or more	22
**Target indications**	**203**
Products in development	101
Approved products	102
Reformulated products	16
Repositioned products	171
Reformulated and repositioned products	16

**Figure 1.**
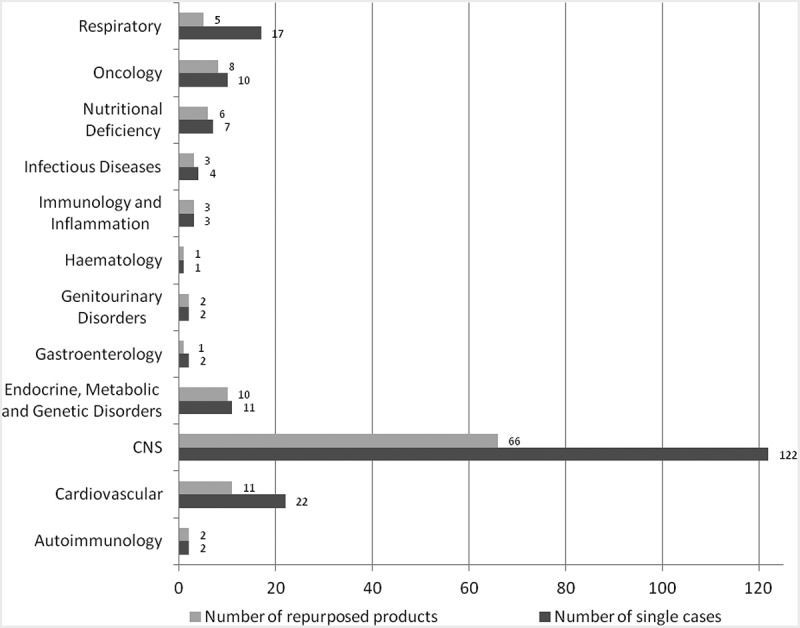
Drugs repurposed into CNS, by initial therapeutic area.

As shown in Figure 1, the highest number of source drugs originated from the CNS therapeutic area (66 out of 118 drugs, 122 single cases – some drugs were repurposed more than once). CNS was followed by cardiovascular (11 drugs, 22 single cases), endocrine, metabolic and genetic disorders (10 drugs, 11 single cases) and oncology (8 drugs, 10 single cases). The remaining 23 products (38 single cases) were distributed evenly among other therapeutic areas. Products sourced from the CNS and cardiovascular areas targeted both neurological and psychiatric disorders, while products from endocrine, metabolic and genetic areas, as well as from oncology, targeted mainly neurological diseases.

**Figure 2.**
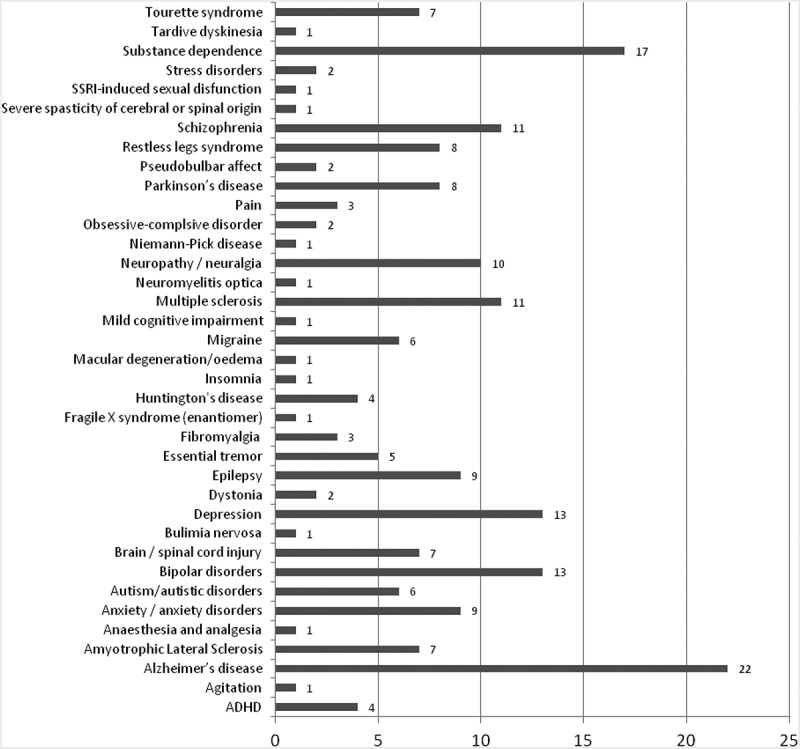
Drug repurposed into CNS, by target indication.

Among new therapeutic indications, Alzheimer’s disease was targeted most often (22 cases), followed by substance dependence (alcohol, drugs/opioids, tobacco), bipolar disorder, depression, neuropathy/neuralgia, multiple sclerosis and schizophrenia, with 10 and more cases each (Figure 2).

**Figure 3.**
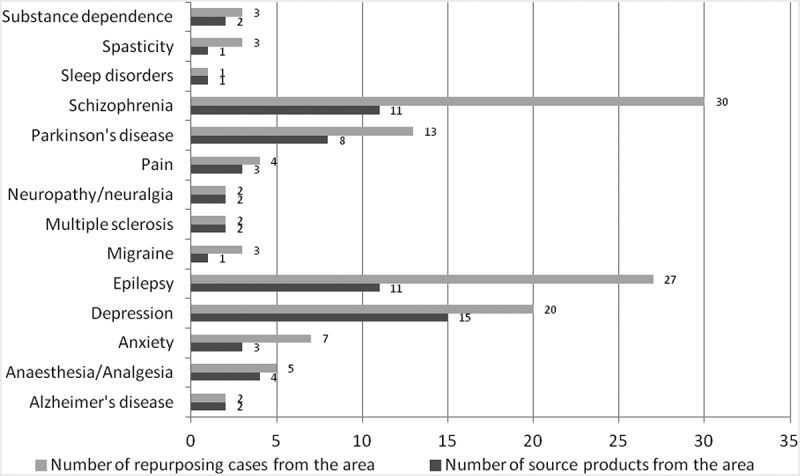
Initial therapeutic indication of drugs repurposed within CNS.

Regarding repurposing within the CNS area, epilepsy, schizophrenia and depression were the richest sources of repurposed drugs with 10 and more products each, targeting 27, 30 and 20 new indications respectively (Figure 3).

## Discussion

We have been able to identify 118 source products targeting 203 new indications.

When analysing approved cases it can be seen that the majority of source products (80%, 82 out of 103 cases) originated from the CNS area. In contrast, amongst products in development, the proportion of CNS source products is much lower (40%, 40 out of 100 cases). For approved products, the high prevalence of CNS-origin drugs may be related to serendipity and a history of clinical experience-driven development within the therapy area. On the other hand, amongst products in development, the increase in the share of ex-CNS drugs repositioned into the area may be related to pharmacology knowledge gained in recent decades, allowing the discovery of new targets for existing products and new aspects of disease pathophysiology outside of the CNS field. However, it is unknown whether this shift in the origin of repurposed drugs means that repurposing in the CNS area is currently adopting a greater range of products to incorporate more non-CNS source drugs, or if the success ratio for approval is higher for drugs sourced within CNS, compared with drugs repurposed from outside the CNS area. Further research will be necessary to track the success rate in terms of regulatory approval for products in development retrieved from our research, and to shed light on whether the regulatory approval rates are indeed higher when products repositioned into CNS are sourced within the same area.

It is noteworthy that about one third of the source products have been repurposed more than once (i.e. have two or more indications approved or in development). This indicates that multiple repurposing for the same source product is not uncommon in the CNS area. Interestingly, the source drugs that underwent multiple repurposing episodes mostly originated from therapeutic areas within CNS (56% of source products repurposed twice and 77% of source products repurposed three times or more). This is not unexpected, as many diseases of the CNS share part of the known pathophysiology (e.g. partial overlap of neuro-receptors or neurotransmitters affected by multiple CNS diseases) and many CNS diseases associate with comorbidities that also affect the CNS. This could also be explained by the fact that most pharmacological agents acting on the CNS target symptoms, which are often common to multiple diseases. For example, there are symptoms that overlap between bipolar disorder, schizophrenia and depression. It is also well known that deep brain stimulation therapy used to alleviate Parkinson’s disease may induce transient acute depression which resolves after adjustment of the electrode position.[20,21] This situation can help provide insights on new development pathways (i.e. new target indications for repurposing) derived from the observation of clinical effects of real-life drug utilisation in its source CNS indication.

When evaluating repurposing into CNS by initial therapeutic area, irrespective of the stage of development, the majority of drugs are repurposed from one CNS indication to another (60% of the indications retrieved), with endocrine, metabolic and genetic disorders, cardiovascular diseases and oncology being the ex-CNS therapeutic areas with the highest frequency of repurposing into CNS. From the cardiovascular drugs repurposed into CNS, with the exception of cilostazol and indobufen, all drugs were initially used as antihypertensives, antiarrhythmics, or both. Despite this common background in terms of cardiovascular effect, there is not a single overlap between the target indications (13 distinct indications), which range from psychiatric to neurological disorders, including neurodegenerative disorders such as Parkinson’s or Alzheimer’s disease. This heterogeneity in terms of target CNS indications for products originating from the cardiovascular area may be explained by the fact that, albeit they have a similar clinical effect within the cardiovascular system, those drugs have different mechanisms of action (ranging from beta-blockers, to angiotensin II receptor antagonists, among others). Indeed those receptors are also present in the CNS and may be involved in CNS disorders. As such, it is not surprising that when repurposed into CNS, those drugs have distinct therapeutic effects compared with their original indication.

In terms of target indications, the endocrine, metabolic and genetic drugs repurposed into CNS have all been, apart from one new indication of insulin, repurposed within neurological disorders. The same is observed for the oncology drugs repurposed into CNS, with the exception of tamoxifene, which was repositioned into bipolar disorder and ondansetron repositioned into substance dependence.

Regarding drugs repurposed within CNS, the most common source indications are epilepsy, depression, schizophrenia and Parkinson’s disease, in total accounting for nearly three quarters (74%) of the cases repositioned within CNS. This result is not unexpected, as those indications are among the most prevalent diseases, each with numerous treatment alternatives; however, there is still very limited knowledge regarding the aetiology and/or pathophysiology of these diseases.

Our research for cases of drug repurposing in the CNS area had the objective to be as extensive and wide as possible, so that a thorough analysis of drug repurposing in CNS could be made possible for the first time. Although we were able to collect both approved and in-development drug repurposing cases in the CNS area, it is expected that more cases may exist that have not been captured even through systematic research, as they may not have been communicated for multiple reasons, including risk of intellectual propriety challenges. Nevertheless, the sheer quantity of products and the number of indications retrieved for drug repurposing in CNS through our research can be considered almost exhaustive, and is sufficient to conclude that drug repurposing is an important development strategy within this currently under-served therapeutic area.

It has to be noted that, as we wanted to identify cases that have meaningful impact on treatment and bring substantial benefit to patients, we excluded minor reformulations that did not lead to changes in the route of administration. We think that inclusion of minor reformulations would lead to identification of many meaningless cases, and would blur the real picture of the pathways for repurposing in the CNS area.

Drug repurposing is an efficient drug development strategy that could be valuable in promoting development efforts in the CNS area, either by producing historic databases of failed drug repurposing cases that can clear the way for alternative research, or by making potential target databases more readily available, so that they can be used for future research and development. Additionally, given the huge unmet need in this therapeutic area, it would be important to consider the role and regulation of market access evaluations in the availability or restriction to patients of medicines issued by repurposing, in order to incentivise value-added drug repurposing in CNS.

The importance of repurposing was recently recognised by the European Commission, which raised the discussion on this topic and constituted the Commission Expert Group on Safe and Timely Access to Medicines for Patients (‘STAMP’). STAMP aims at recognizing the importance of comprehensive investigation of different opportunities that a molecule could bring to patients, with faster development times, and at reduced costs and risk for pharmaceutical companies.[22]

Several initiatives focusing on promoting drug repurposing and based on public, private and academic partnership have also been established. In the UK in 2011, the Medical Research Council established a partnership with AstraZeneca to give academic researchers access to clinical and preclinical compounds for potential repurposing.[23] In France, the National Cancer Institute (INCa), in agreement with the French National Agency for Medicines and Health Products Safety (ANSM), launched the AcSé program in 2013. The program aims at securing access to innovative targeted therapies for cancer patients who failed with validated therapies. The AcSé crizotinib project was the first clinical trial of the AcSé program.[24] In the USA, the Discovering New Therapeutic Uses for Existing Molecules (New Therapeutic Uses) program was initiated in 2012 by the National Center for Advancing Translational Sciences, Pfizer, AstraZeneca, and Eli Lilly. The program matches researchers with a selection of pharmaceutical industry compounds to help scientists explore new treatment options.[25,26] Thus, drug repurposing is gaining more and more interest worldwide. This is comforting, as repurposing may be a gold mine for future CNS drug development and should be strongly considered by drug developers.

## Conclusion

In the research conducted, we were able to find 203 target indications in the CNS area, where drugs were developed through repurposing (mainly repositioning) from 118 source products. This means that drug repurposing in CNS is a relevant and currently very active drug development method, exemplified by the considerable number of new indications that have been found via this strategy, with approximately half of the target indications currently under development.

Public policies that support and encourage the development of new CNS therapies may prove critical to bringing new therapeutic options to the market for a currently very poorly serviced population. Developing incentives for manufacturers to systematically explore repurposing may significantly help to address the high unmet need amongst patients suffering from CNS disorders.

## Supplementary Material

Supplementary Material
