# Supplementary material for: Filling the gap in CNS drug development: evaluation of the role of drug repurposing
Source: J Mark Access Health Policy. 2017 Apr 10;5(1):1299833. doi: 10.1080/20016689.2017.1299833 (PMC5405562; doi:10.1080/20016689.2017.1299833)
Supplement: Supplementary Material [file zjma_a_1299833_sm4893.docx]

Supplementary Material

Table 1. Search strategy used in PubMed and EMBASE databases.

| **No** | **Query** |
| --- | --- |
| 1 | Brain damage.mp. [mp=ti, ab, hw, tn, ot, dm, mf, dv, kw, nm, kf, px, rx, ui] |
| 2 | Brain dysfunction.mp. [mp=ti, ab, hw, tn, ot, dm, mf, dv, kw, nm, kf, px, rx, ui] |
| 3 | Spinal cord.mp. [mp=ti, ab, hw, tn, ot, dm, mf, dv, kw, nm, kf, px, rx, ui] |
| 4 | (disorder or pathology or injury or inflammation).mp. [mp=ti, ab, hw, tn, ot, dm, mf, dv, kw, nm, kf, px, rx, ui] |
| 5 | 3 and 4 |
| 6 | Seizure.mp. [mp=ti, ab, hw, tn, ot, dm, mf, dv, kw, nm, kf, px, rx, ui] |
| 7 | epilepsy.mp. [mp=ti, ab, hw, tn, ot, dm, mf, dv, kw, nm, kf, px, rx, ui] |
| 8 | (Parkinson's disease or Essential tremor or Amyotrophic lateral sclerosis or Tourette's Syndrome or Multiple sclerosis).mp. [mp=ti, ab, hw, tn, ot, dm, mf, dv, kw, nm, kf, px, rx, ui] |
| 9 | (Sleep disorder or Narcolepsy).mp. [mp=ti, ab, hw, tn, ot, dm, mf, dv, kw, nm, kf, px, rx, ui] |
| 10 | Migraine.mp. [mp=ti, ab, hw, tn, ot, dm, mf, dv, kw, nm, kf, px, rx, ui] |
| 11 | Neuropathic pain.mp. [mp=ti, ab, hw, tn, ot, dm, mf, dv, kw, nm, kf, px, rx, ui] |
| 12 | (Attention deficit hyperactivity disorder or ADHD or Autism or Obsessive compulsive disorder).mp. [mp=ti, ab, hw, tn, ot, dm, mf, dv, kw, nm, kf, px, rx, ui] |
| 13 | (Huntington's disease or Alzheimer's disease).mp. [mp=ti, ab, hw, tn, ot, dm, mf, dv, kw, nm, kf, px, rx, ui] |
| 14 | dementia.mp. [mp=ti, ab, hw, tn, ot, dm, mf, dv, kw, nm, kf, px, rx, ui] |
| 15 | stroke.mp. [mp=ti, ab, hw, tn, ot, dm, mf, dv, kw, nm, kf, px, rx, ui] |
| 16 | 1 or 2 or 5 or 6 or 7 or 8 or 9 or 10 or 11 or 12 or 13 or 14 or 15 |
| 17 | Alcohol abuse.mp. [mp=ti, ab, hw, tn, ot, dm, mf, dv, kw, nm, kf, px, rx, ui] |
| 18 | Alcohol dependence.mp. [mp=ti, ab, hw, tn, ot, dm, mf, dv, kw, nm, kf, px, rx, ui] |
| 19 | Anorexia nervosa.mp. [mp=ti, ab, hw, tn, ot, dm, mf, dv, kw, nm, kf, px, rx, ui] |
| 20 | Anxiety.mp. [mp=ti, ab, hw, tn, ot, dm, mf, dv, kw, nm, kf, px, rx, ui] |
| 21 | Bipolar disorder.mp. [mp=ti, ab, hw, tn, ot, dm, mf, dv, kw, nm, kf, px, rx, ui] |
| 22 | Bulimia nervosa.mp. [mp=ti, ab, hw, tn, ot, dm, mf, dv, kw, nm, kf, px, rx, ui] |
| 23 | schizophrenia.mp. [mp=ti, ab, hw, tn, ot, dm, mf, dv, kw, nm, kf, px, rx, ui] |
| 24 | Cognitive disorder.mp. [mp=ti, ab, hw, tn, ot, dm, mf, dv, kw, nm, kf, px, rx, ui] |
| 25 | Depressive disorder.mp. [mp=ti, ab, hw, tn, ot, dm, mf, dv, kw, nm, kf, px, rx, ui] |
| 26 | insomnia.mp. [mp=ti, ab, hw, tn, ot, dm, mf, dv, kw, nm, kf, px, rx, ui] |
| 27 | Manic episode.mp. [mp=ti, ab, hw, tn, ot, dm, mf, dv, kw, nm, kf, px, rx, ui] |
| 28 | Mixed episode.mp. [mp=ti, ab, hw, tn, ot, dm, mf, dv, kw, nm, kf, px, rx, ui] |
| 29 | Nicotine withdrawal.mp. [mp=ti, ab, hw, tn, ot, dm, mf, dv, kw, nm, kf, px, rx, ui] |
| 30 | phobia.mp. [mp=ti, ab, hw, tn, ot, dm, mf, dv, kw, nm, kf, px, rx, ui] |
| 31 | agitation.mp. [mp=ti, ab, hw, tn, ot, dm, mf, dv, kw, nm, kf, px, rx, ui] |
| 32 | irritability.mp. [mp=ti, ab, hw, tn, ot, dm, mf, dv, kw, nm, kf, px, rx, ui] |
| 33 | depression.mp. [mp=ti, ab, hw, tn, ot, dm, mf, dv, kw, nm, kf, px, rx, ui] |
| 34 | Restless legs syndrome.mp. [mp=ti, ab, hw, tn, ot, dm, mf, dv, kw, nm, kf, px, rx, ui] |
| 35 | (CNS or central nervous system).mp. [mp=ti, ab, hw, tn, ot, dm, mf, dv, kw, nm, kf, px, rx, ui] |
| 36 | 16 or 17 or 18 or 19 or 20 or 21 or 22 or 23 or 24 or 25 or 26 or 27 or 28 or 29 or 30 or 31 or 32 or 33 or 34 or 35 |
| 37 | (repositioning or repurposing or reprofiling or re-profiling or re-tasking or retasking or repositioned or repurposed or reprofiled or re-profiled or retasked or re-tasked).ti,ab. |
| 38 | 36 and 37 |
| 39 | remove duplicates from 38 |
| 40 | limit 39 to english language |
| 41 | limit 40 to human |
| 42 | limit 41 to humans |

Table 2. Drugs Repositioned in CNS

| **No** | **Drug** | **Initial indication** | **Initial therapeutic area** | **New indication** | **Reformulation/ Repositioninging/Both** | **Development status** |
| --- | --- | --- | --- | --- | --- | --- |
| **1** | **Albuterol (24,49)** | Relief of bronchospasm | Respiratory | Brain / spinal cord injury | Reformulation and Repositioning | In development |
| **2** | **Alemtuzumab (59, 65, 72, 85)** | Leukaemia | Oncology | Multiple sclerosis | Repositioning | Approved |
| **3** | **Amantadine (148)** | Influenza | Infectious disease | Parkinson’s disease | Repositioning | Approved |
| **4** | **Apomorphine (35, 36)** | Parkinson’s disease | CNS | Brain / spinal cord injury | Repositioning | In development |
| **5** | **Aripiprazole (105)** | Schizophrenia | CNS | Substance dependence | Repositioning | In development |
| **6** | **Aripiprazole (55, 74)** | Schizophrenia | CNS | Tourette's syndrome | Repositioning | Approved |
| **7** | **Aripiprazole (74)** | Schizophrenia | CNS | Acute agitation (intramuscular) | Reformulation and Repositioning | Approved |
| **8** | **Aripiprazole (74)** | Schizophrenia | CNS | Autism | Repositioning | Approved |
| **9** | **Aripiprazole (74)** | Schizophrenia | CNS | Depression | Repositioning | Approved |
| **10** | **Aripiprazole (74)** | Schizophrenia | CNS | Bipolar disorder | Repositioning | Approved |
| **11** | **Aripiprazole (105)** | Schizophrenia | CNS | Alzheimer’s disease (iv, oral) | Repositioning | In development |
| **12** | **Asenapine (79, 135)** | Schizophrenia | CNS | Bipolar disorder | Repositioning | Approved |
| **13** | **Atomoxetine (4, 80, 143, 148, 156)** | Depression | CNS | ADHD | Repositioning | Approved |
| **14** | **Atorvastatin (3, 157, 160)** | Hypercholesterolemia | Endocrine, Metabolic and Genetic Disorders | Multiple sclerosis | Repositioning | In development |
| **15** | **Baclofene (10, 12, 33)** | Severe spasticity | CNS | Substance dependence (alcohol) | Repositioning | Approved |
| **16** | **Baclofene (10, 12, 75)** | Severe spasticity | CNS | Severe spasticity (intrathecal) | Reformulation | Approved |
| **17** | **Baclofene (10, 12, 88)** | Severe spasticity | CNS | Fragile X syndrome (enantiomer) | Reformulation and Repositioning | In development |
| **18** | **Bevacizumab/Ranibizumab (62, 66, 147)** | Cancer | Oncology | Macular degeneration (injection) | Reformulation and Repositioning | Approved |
| **19** | **Biotin (104, 157)** | Nutritional deficiency | Nutritional deficiency | Multiple sclerosis | Repositioning | In development |
| **20** | **Biperiden (50, 169)** | Parkinson’s disease | CNS | Brain / spinal cord injury | Repositioning | In development |
| **21** | **Brexpiprazole (130)** | Schizophrenia | CNS | Alzheimer’s disease (agitation in Alzheimer’s disease) | Repositioning | In development |
| **22** | **Bumetanide (37, 153)** | Hypertension (oedema, diuretic) | Cardiovascular | Autism (in children) | Repositioning | In development |
| **23** | **Bupivacaineb (8)** | Anaesthesia / analgesia | CNS | Neuropathy / neuralgia (post herpetic neuralgia) | Reformulation and Repositioning | In development |
| **24** | **Buprenorphine (156)** | Pain (analgesia) | CNS | Substance dependence | Repositioning | Approved |
| **25** | **Bupropion (4, 142, 148, 156, 159)** | Depression | CNS | Substance dependence | Repositioning | Approved |
| **26** | **Buspirone (26, 93, 149, 167)** | Anxiety | CNS | Substance dependence | Repositioning | In development |
| **27** | **Buspirone (25)** | Anxiety | CNS | Autism (in children) | Repositioning | In development |
| **28** | **Buspirone (+levodopa) (131)** | Anxiety | CNS | Brain / spinal cord injures | Repositioning | In development |
| **29** | **Cabergoline (10)** | Parkinson’s disease/ anti-hyperprolactinemic | CNS | Restless legs syndrome | Repositioning | In development |
| **30** | **Caffeine (10)** | Apnoea | Respiratory | Stress disorders | Repositioning | Approved |
| **31** | **Caffeine (52)** | Apnoea | Respiratory | Parkinson’s disease | Repositioning | In development |
| **32** | **Capsaicin (17)** | Herpetic neuralgia | CNS | Neuropathy / neuralgia (HIV-associated neuropathy) | Repositioning | In development |
| **33** | **Carbamazepine (100)** | Epilepsy (oral) | CNS | Epilepsy (intravenous) | Reformulation | In development |
| **34** | **Carbamazepine (135)** | Epilepsy | CNS | Bipolar disorder | Repositioning | Approved |
| **35** | **Carbamazepine (106, 111, 124)** | Epilepsy | CNS | Neuropathy / neuralgia | Repositioning | Approved |
| **36** | **Ceftriaxone sodium (53, 94)** | Infections (antibiotic) | Infectious disease | Amyotrophic lateral sclerosis | Repositioning | In development |
| **37** | **Cerebrolysin (91, 135)** | Alzheimer disease | CNS | Brain / spinal cord injury | Repositioning | Approved |
| **38** | **Chlorpromazine (4, 156)** | Antiemetic/antihistamine | Gastroenterology | Anxiety / anxiety disorders | Repositioning | Approved |
| **39** | **Chlorpromazine (4, 156)** | Antiemetic/antihistamine | Gastroenterology | Schizophrenia | Repositioning | Approved |
| **40** | **Cilostazol (9, 27)** | Intermittent claudication | Cardiovascular | Alzheimer’s Disease | Repositioning | In development |
| **41** | **Citalopram/ Escitalopram (92, 142)** | Depression | CNS | Depression | Reformulation | Approved |
| **42** | **Clobazam (166)** | Anxiety, epilepsy | CNS | Epilepsy (Lennox-Gastaut syndrome) | Repositioning | Approved |
| **43** | **Clonazepam (118)** | Epilepsy | CNS | Epilepsy (intranasal spray) | Reformulation | In development |
| **44** | **Clonidine (113, 115)** | Hypertension | Cardiovacular | Substance dependence (opioid) | Repositioning | Approved |
| **45** | **Clonidine (114)** | Hypertension | Cardiovacular | ADHD | Repositioning | In development |
| **46** | **Clonidine (112)** | Hypertension | Cardiovacular | Migraine | Repositioning | Approved |
| **47** | **Coenzyme Q10 (10)** | Nutritional deficiency | Nutritional deficiency | Huntington’s disease | Repositioning | In development |
| **48** | **Creatine (10)** | Nutritional deficiency | Nutritional deficiency | Parkinson’s disease | Repositioning | In development |
| **49** | **Creatine (21, 28, 29, 30)** | Nutritional deficiency | Nutritional deficiency | Amyotrophic lateral sclerosis | Repositioning | In development |
| **50** | **Cyanocobalamine (vitamin B12) (8)** | Nutritional deficiency | Nutritional deficiency | Pain | Repositioning | Approved |
| **51** | **Cyclosporine (2)** | Prophylaxis of organ rejection | Immunology and Inflammation | Amyotrophic lateral sclerosis | Reformulation and Repositioning | In development |
| **52** | **Cysteamine hydrochloride (31, 43)** | Nephropathic cystinosis | Endocrine, Metabolic and Genetic Disorders | Huntington's disease | Repositioning | In development |
| **53** | **Daclizumab (70, 140)** | Acute organ rejection | Immunology and Inflammation | Multiple sclerosis | Repositioning | In development |
| **54** | **Dextromethorphan (+quinidine) (5, 67, 127)** | Opioid cough suppressant (+arrhythmia) | Respiratory | Pseudobular affect | Repositioning | Approved |
| **55** | **Dextromethorphan (+quinidine) (5, 67, 127)** | Opioid cough suppressant (+arrhythmia) | Respiratory | Amyotrophic lateral sclerosis | Repositioning | In development |
| **56** | **Dextromethorphan (+quinidine) (5, 67, 127)** | Opioid cough suppressant (+arrhythmia) | Respiratory | Depression (treatment resistant) | Repositioning | In development |
| **57** | **Dextromethorphan (+quinidine) (5, 67, 127)** | Opioid cough suppressant (+arrhythmia) | Respiratory | Autism | Repositioning | In development |
| **58** | **Dextromethorphan (+quinidine) (5, 67, 127)** | Opioid cough suppressant (+arrhythmia) | Respiratory | Alzheimer’s disease (agitation) | Repositioning | In development |
| **59** | **Dextromethorphan (+quinidine) (5, 67, 127)** | Opioid cough suppressant (+arrhythmia) | Respiratory | Parkinson's disease (levodopa induced dyskinesia) | Repositioning | In development |
| **60** | **Dextromethorphan (+ quinidine) (127, 164)** | Opioid cough suppressant (+arrhythmia) | Respiratory | Multiple sclerosis | Repositioning | In development |
| **61** | **Dextromethorphan (+ quinidine) (5, 127)** | Opioid cough suppressant (+arrhythmia) | Respiratory | Schizophrenia | Repositioning | In development |
| **62** | **Dihydroergocryptine (135)** | Migraine disorders | CNS | Depression | Repositioning | Approved |
| **63** | **Dihydroergocryptine (135)** | Migraine disorders | CNS | Parkinson’s disease | Repositioning | Approved |
| **64** | **Dihydroergocryptine (135)** | Migraine disorders | CNS | Alzheimer’s disease | Repositioning | Approved |
| **65** | **Dimethyl fumarate (71, 151)** | Psoriasis | Autoimmunology | Multiple sclerosis | Repositioning | Approved |
| **66** | **Doxepin (142, 159)** | Depression (major depressive disorder) | CNS | Insomnia | Repositioning | Approved |
| **67** | **Duloxetine (99, 141, 142, 159)** | Depression (major depressive disorder) | CNS | Anxiety / anxiety disorders (general anxiety disorder) | Repositioning | Approved |
| **68** | **Duloxetine (99, 141, 142, 159)** | Depression (major depressive disorder) | CNS | Pain (chronic musculoskeletal) | Repositioning | Approved |
| **69** | **Duloxetine (99, 141, 142, 159)** | Depression (major depressive disorder) | CNS | Fibromyalgia | Repositioning | Approved |
| **70** | **Eculizumab (96, 101)** | Atypical hemolytic-uremic syndrome/paroxysmal nocturnal hemoglobinuria | Hematology | Neuromyelitis optica | Repositioning | In development |
| **71** | **Etanercept (14,156, 162)** | Rheumatoid arthritis | Autoimmunology | Alzheimer’s disease | Repositioning | In development |
| **72** | **Everolimus (108)** | Cancer | Oncology | Epilepsy | Repositioning | In development |
| **73** | **Exenatide (22)** | Diabetes mellitus | Endocrine, Metabolic and Genetic Disorders | Parkinson’s Disease | Repositioning | In development |
| **74** | **Fenfluramine (171)** | Obesity (withdrawn) | Endocrine, Metabolic and Genetic Disorders | Epilepsy (Dravet syndrome) | Repositioning | In development |
| **75** | **Fentanyl (68)** | Pain (cancer pain, tablet) | CNS | Pain (cancer pain, nasal spray) | Reformulation | Approved |
| **76** | **Fluoxetine (135)** | Depression | CNS | Bipolar disorder | Repositioning | Approved |
| **77** | **Fluoxetine (135)** | Depression | CNS | Obsessive-compulsive disorder | Repositioning | Approved |
| **78** | **Fluoxetine (135)** | Depression | CNS | Bulimia Nervosa | Repositioning | Approved |
| **79** | **Fluvoxamine (145)** | Depression | CNS | Obsessive-compulsive disorder | Repositioning | Approved |
| **80** | **Fluvoxamine (145)** | Depression | CNS | Anxiety / anxiety disorders (social anxiety disorder) | Repositioning | Approved |
| **81** | **Gabapentin (102)** | Epilepsy | CNS | Substance dependence (alcohol) | Repositioning | In development |
| **82** | **Gabapentin (10, 76, 156)** | Epilepsy | CNS | Anxiety / anxiety disorders | Repositioning | In development |
| **83** | **Gabapentin (10, 76, 156)** | Epilepsy | CNS | Neuropathy / neuralgia | Repositioning | Approved |
| **84** | **Gabapentin (10, 76, 156)** | Epilepsy | CNS | Restless legs syndrome | Repositioning | Approved |
| **85** | **Galantamine (4, 139, 141)** | Polio paralysis and neuropathic pain | CNS | Mild to moderate dementia of the Alzheimer’s type | Repositioning | Approved |
| **86** | **Guanfacine (10)** | Hypertension | Cardiovascular | ADHD | Repositioning | Approved |
| **87** | **Guanfacine (10)** | Hypertension | Cardiovascular | Tourette syndrome | Repositioning | In development |
| **88** | **Haloperidol (136)** | Schizophrenia (oral) | CNS | Schizophrenia (intramuscular) | Reformulation | Approved |
| **89** | **Haloperidol (10)** | Schizophrenia | CNS | Tourette syndrome | Repositioning | Approved |
| **90** | **Ibudilast (6)** | Asthma | Respiratory | Substance dependence (alcohol) | Repositioning | In development |
| **91** | **Ibudilast (6)** | Asthma | Respiratory | Substance dependence (opioid) | Repositioning | In development |
| **92** | **Ibudilast (119)** | Asthma | Respiratory | Amyotrophic lateral sclerosis | Repositioning | In development |
| **93** | **Ibudilast (119)** | Asthma | Respiratory | Substance dependence (methamphetamine) | Repositioning | In development |
| **94** | **Ibudilast (119)** | Asthma | Respiratory | Multiple sclerosis | Repositioning | In development |
| **95** | **Immunoglobulin, intravenous Immunoglobulin G, human (3)** | Anti-inflammatory | Immunology and Inflammation | Alzheimer’s disease | Repositioning | In development |
| **96** | **Indobufen (135)** | Thromboembolic disorders/Stroke | Cardiovascular | Migraine disorders | Repositioning | Approved |
| **97** | **Insulin (54)** | Diabetes mellitus | Endocrine, Metabolic and Genetic Disorders | Depression (major depressive disorders, intranasal) | Reformulation and Repositioning | In development |
| **98** | **Insulin (117)** | Diabetes mellitus | Endocrine, Metabolic and Genetic Disorders | Alzheimer’s disease (intranasal) | Reformulation and Repositioning | In development |
| **99** | **Interferon beta-1a (77)** | Multiple sclerosis | CNS | Multiple sclerosis (pegylation) | Reformulation | Approved |
| **100** | **Ketamine (1, 103, 150)** | Anaesthesia / analgesia | CNS | Anaesthesia / analgesia (enantiomer) | Reformulation | Approved |
| **101** | **Ketamine (1, 90, 103, 150)** | Anaesthesia / analgesia | CNS | Depression (major depressive disorder) | Repositioning | In development |
| **102** | **Lamotrigine (81)** | Epilepsy | CNS | Bipolar disorder | Repositioning | Approved |
| **103** | **Lamotrigine (120)** | Epilepsy | CNS | Schizophrenia | Repositioning | In development |
| **104** | **Levetiracetam (109)** | Epilepsy (partial seizures) | CNS | Mild cognitive impairment | Repositioning | In development |
| **105** | **Levodopa (128)** | Parkinson’s disease | CNS | Restless legs syndrome | Repositioning | Approved |
| **106** | **Levodopa (51, 152)** | Parkinson’s disease | CNS | Schizophrenia | Repositioning | In development |
| **107** | **Levodopa + (buspirone) (131)** | Parkinson’s disease | CNS | Brain / spinal cord injures | Repositioning | In development |
| **108** | **Lidocaine (8, 121)** | Anaesthesia/ arrhythmia (gel, cream, injection) | CNS | Neuropathy / neuralgia (post-herpetic, transdermal patch) | Reformulation and Repositioning | Approved |
| **109** | **Liraglutide (38, 95)** | Diabetes mellitus | Endocrine, Metabolic and Genetic Disorders | Alzheimer’s Disease | Repositioning | In development |
| **110** | **Lisuride (10, 135)** | Parkinson’s disease | CNS | Migraine disorders | Repositioning | Approved |
| **111** | **L-methylfolate (116)** | Nutritional deficiency | Nutritional deficiency | Resistant depression | Repositioning | Approved |
| **112** | **Loxapine (61)** | Schizophrenia (caps) | CNS | Schizophrenia (agitation) (inhalation powder) | Reformulation | Approved |
| **113** | **Loxapine (61)** | Schizophrenia (caps) | CNS | Bipolar disorder (agitation) (inhalation powder) | Reformulation and Repositioning | Approved |
| **114** | **Masitinib (18, 157, 158)** | Oncology | Oncology | Multiple sclerosis | Repositioning | In development |
| **115** | **Masitinib (123)** | Oncology | Oncology | Alzheimer’s Disease | Repositioning | In development |
| **116** | **Masitinib (123)** | Oncology | Oncology | Amyotrophic lateral sclerosis | Repositioning | In development |
| **117** | **Memantine (156)** | Influenza | Infectious disease | Alzheimer’s disease | Repositioning | Approved |
| **118** | **Memantine (83, 97)** | Influenza | Infectious disease | Schizophrenia | Repositioning | In development |
| **119** | **Metformin (11, 32, 41, 87)** | Diabetes mellitus | Endocrine, Metabolic and Genetic Disorders | Alzheimer’s Disease | Repositioning | In development |
| **120** | **Methylene blue (3, 122)** | Methemoglobinemia (injection) | Endocrine, Metabolic and Genetic Disorders | Alzheimer’s disease (tablet) | Reformulation and Repositioning | In development |
| **121** | **Midazolam (134)** | Anxiety | CNS | Epilepsy (acute repetitive seizures, nasal spray) | Reformulation and Repositioning | In development |
| **122** | **Midazolam (63)** | Anxiety | CNS | Epilepsy (seizure clusters, oromucosal solution) | Reformulation and Repositioning | Approved |
| **123** | **Midazolam (137)** | Anxiety (oral) | CNS | Anxiety / anxiety disorders (parenteral) | Reformulation | Approved |
| **124** | **Mifepristone (42)** | Termination of pregnancy | Genitourinary disorders | Substance dependence (alcohol) | Repositioning | In development |
| **125** | **Miglustat (69)** | Mild to moderate Type I Gaucher disease | Endocrine, Metabolic and Genetic Disorders | Neurological manifestations of Niemann-Pick disease, type C | Repositioning | Approved |
| **126** | **Milnacipran (8)** | Antidepressant | CNS | Fibromyalgia | Repositioning | Approved |
| **127** | **Milnacipran/levomilnacipran (89)** | Major depressive disorder | CNS | Depression (major depressive disorder, enantiomer) | Reformulation | Approved |
| **128** | **Mirtazapine (13, 39, 161)** | Depression | CNS | Schizophrenia | Repositioning | In development |
| **129** | **Moclobemide (126)** | Depression | CNS | Anxiety / anxiety disorders (social phobia) | Repositioning | Approved |
| **130** | **Nabiximols (135)** | Multiple sclerosis | CNS | Neuropathy / neuralgia | Repositioning | In development |
| **131** | **N-acetylcysteine (+naltrexone) (44)** | Mucolytic | Respiratory | Substance dependence (alcohol) | Repositioning | In development |
| **132** | **Nadolol (10)** | Hypertension, arrhythmia (brak rejestracji na EMA) | Cardiovascular | Essential tremor | Repositioning | Approved |
| **133** | **Naloxone (+oxycodone) (10)** | Substance dependence (Opioid abuse, injection) | CNS | Restless legs syndrome (tablet) | Reformulation and Repositioning | Approved |
| **134** | **Naltrexone (+N-acetylcysteione) (44)** | Opioid | CNS | Substance dependence (alcohol) | Repositioning | In development |
| **135** | **Naltrexone (159)** | Substance dependence (opioid and alcohol, tabl.) | CNS | Substance dependence (opioid and alcohol, injection) | Reformulation | Approved |
| **136** | **Nilvadipine (3, 20, 57, 58, 95)** | Hypertension (tablet) | Cardiovascular | Alzheimer’s disease (tablet) | Repositioning | In development |
| **137** | **Ofatumumab (45, 84)** | Oncology | Oncology | Multiple sclerosis | Repositioning | In development |
| **138** | **Olanzapine (10, 135)** | Schizophrenia | CNS | Depression | Repositioning | Approved |
| **139** | **Olanzapine (10, 135)** | Schizophrenia | CNS | Bipolar disorder | Repositioning | Approved |
| **140** | **Olanzapine (10, 135)** | Schizophrenia | CNS | Huntington’s disease | Repositioning | In development |
| **141** | **Ondansetron (107)** | Chemotherapy Induced Nausea and Vomiting | Oncology | Substance dependence (Alcohol) | Repositioning | In development |
| **142** | **Oxcarbazepine (46)** | Epilepsy | CNS | Neuropathy / neuralgia | Repositioning | In development |
| **143** | **Oxycodone (+naloxone) (10)** | Pain/analgesia (tablet) | CNS | Restless legs syndrome (tablet) | Repositioning | Approved |
| **144** | **Paroxetine (146)** | Depression | CNS | Anxiety / anxiety disorders | Repositioning | Approved |
| **145** | **Phenobarbital (135)** | Sleep disorders | CNS | Epilepsy | Repositioning | Approved |
| **146** | **Pimozide (10)** | Antypsychotic | CNS | Tourette syndrome | Repositioning | In development |
| **147** | **Pioglitazone (3,82)** | Diabetes mellitus | Endocrine, Metabolic and Genetic Disorders | Alzheimer’s disease | Repositioning | In development |
| **148** | **Pramipexole (10, 135, 139, 156)** | Parkinson’s disease | CNS | Restless legs syndrome | Repositioning | Approved |
| **149** | **Pramipexole (10, 135, 139, 156)** | Parkinson’s disease | CNS | Essential tremor | Repositioning | In development |
| **150** | **Pramipexole (7,10, 135, 139, 156)** | Parkinson’s disease | CNS | Depression | Repositioning | In development |
| **151** | **Prazosin (47)** | Hypertension | Cardiovascular | Substance dependence (alcohol) | Repositioning | In development |
| **152** | **Pregabalin (10, 135, 156)** | Epilepsy (Partial seizures) | CNS | Neuropathy / neuralgia | Repositioning | Approved |
| **153** | **Pregabalin (10, 135, 156)** | Epilepsy (Partial seizures) | CNS | Anxiety / anxiety disorders | Repositioning | Approved |
| **154** | **Pregabalin (10, 135, 156)** | Epilepsy (Partial seizures) | CNS | Fibromyalgia | Repositioning | Approved |
| **155** | **Primidone (10)** | Epilepsy | CNS | Essential tremor | Repositioning | Approved |
| **156** | **Progesterone (34,73)** | Amenorrhea and abnormal uterine bleeding | Genitourinary disorders | Brain / spinal cord injury (traumatic brain injury) | Reformulation and Repositioning | In development |
| **157** | **Propranolol (141, 148, 156)** | Hypertension, arrhytmia | Cardiovascular | Migraine | Repositioning | Approved |
| **158** | **Propranolol (141, 148, 156)** | Hypertension, arrhytmia | Cardiovascular | Essential tremor | Repositioning | Approved |
| **159** | **Quetiapine (135)** | Schizophrenia | CNS | Resistant depression | Repositioning | Approved |
| **160** | **Quetiapine (135)** | Schizophrenia | CNS | Bipolar disorder | Repositioning | Approved |
| **161** | **Quinidine (+dextromethorphan) (5, 67, 127)** | Arrhythmia (+cough suppressant) | Cardiovascular | Pseudobulbar affect | Repositioning | Approved |
| **162** | **Quinidine (+dextromethorphan) (5, 67, 127)** | Arrhythmia (+cough suppressant) | Cardiovascular | Amyotrophic lateral sclerosis | Repositioning | In development |
| **163** | **Quinidine (+dextromethorphan) (5, 67, 127)** | Arrhythmia (+cough suppressant) | Cardiovascular | Resistant depression | Repositioning | In development |
| **164** | **Quinidine (+dextromethorphan) (5, 67, 127)** | Arrhythmia (+cough suppressant) | Cardiovascular | Autism | Repositioning | In development |
| **165** | **Quinidine (+dextromethorphan) (5, 67, 127)** | Arrhythmia (+cough suppressant) | Cardiovascular | Parkinson's disease (Levodopa induced dyskinesia) | Repositioning | In development |
| **166** | **Quinidine (+dextromethorphan) (5, 67, 127)** | Arrhythmia (+cough suppressant) | Cardiovascular | Alzheimer’s disease | Repositioning | In development |
| **167** | **Quinidine (+dextromethorphan) (5, 67, 127)** | Arrhythmia (+cough suppressant) | Cardiovascular | Schizophrenia | Repositioning | In development |
| **168** | **Quinidine (+dextromethorphan) (5, 67, 127)** | Arrhythmia (+cough suppressant) | Cardiovascular | Multiple sclerosis | Repositioning | In development |
| **169** | **Risperidone (10, 135, 142)** | Schizophrenia | CNS | Bipolar disorder (LAI) | Reformulation and Repositioning | Approved |
| **170** | **Risperidone (10, 135, 142)** | Schizophrenia | CNS | Autism | Repositioning | Approved |
| **171** | **Risperidone (10, 135, 142)** | Schizophrenia | CNS | Alzheimer’s disease (agitation) | Repositioning | Approved |
| **172** | **Risperidone (10, 135, 142,154)** | Schizophrenia | CNS | Tourette syndrome | Repositioning | In development |
| **173** | **Risperidone (10, 135, 142)** | Schizophrenia | CNS | Bipolar Disorders | Repositioning | Approved |
| **174** | **Risperidone (78)** | Schizophrenia (oral) | CNS | Schizophrenia (injection) | Reformulation | Approved |
| **175** | **Risperidone/ paliperidone (10, 135, 142)** | Schizophrenia | CNS | Schizophrenia (Paliperidone - active metabolite of risperidone) | Reformulation | Approved |
| **176** | **Rivastigmine (64)** | Alzheimer’s disease | CNS | Dementia in Alzheimer’s disease (patch) | Reformulation | Approved |
| **177** | **Ropinirole (10, 135, 139, 148, 156)** | Parkinson’s disease | CNS | Restless legs syndrome | Repositioning | Approved |
| **178** | **Ropinirole (10, 135, 139, 148, 156)** | Parkinson’s disease | CNS | SSRI-induced sexual dysfunction | Repositioning | In development |
| **179** | **Rotigotine (10, 135)** | Parkinson’s disease | CNS | Restless legs syndrome | Repositioning | Approved |
| **180** | **S-Adenosyl methionine (98,138)** | Nutritional deficiency | Nutritional deficiency | Depression | Repositioning | In development |
| **181** | **Saracatinib (19, 144)** | Oncology | Oncology | Alzheimer’s Disease | Repositioning | In development |
| **182** | **Sertraline (163)** | Depression | CNS | Anxiety / anxiety disorders | Repositioning | Approved |
| **183** | **Tamoxifen (156, 170, 171)** | Breast cancer | Oncology | Bipolar disorder | Repositioning | In development |
| **184** | **Telmisartan (48, 165)** | Hypertension | Cardiovascular | Alzheimer’s disease | Repositioning | In development |
| **185** | **Tetrabenazine (23, 132)** | Schizophrenia | CNS | Tourette's Syndrome in children | Repositioning | Approved |
| **186** | **Tetrabenazine (10, 135)** | Schizophrenia | CNS | Dystonia | Repositioning | Approved |
| **187** | **Tetrabenazine (10, 135)** | Schizophrenia | CNS | Huntington disease | Repositioning | Approved |
| **188** | **Tetrabenazine (132)** | Schizophrenia | CNS | Tardive dyskinesia | Repositioning | Approved |
| **189** | **Topiramate (10, 143)** | Epilepsy | CNS | Substance dependence (alcohol and cocaine) | Repositioning | In development |
| **190** | **Topiramate (10, 143)** | Epilepsy | CNS | Migraine | Repositioning | Approved |
| **191** | **Topiramate (10, 16, 143)** | Epilepsy | CNS | Essential tremor | Repositioning | In development |
| **192** | **Topiramate (10, 143, 168)** | Epilepsy | CNS | Tourette syndrome | Repositioning | In development |
| **193** | **Topiramate (110)** | Epilepsy | CNS | Bipolar disorders | Repositioning | Approved |
| **194** | **Topiramate (129)** | Epilepsy | CNS | Stress disorders (post-traumatic) | Repositioning | Approved |
| **195** | **Tramadol hydrochloride (60, 56)** | Pain (moderate to moderately severe) | CNS | Neuropathy / neuralgia (post-herpetic neuralgia) | Repositioning | In development |
| **196** | **Tramadol hydrochloride (60, 56)** | Pain (moderate to moderately severe) | CNS | Neuropathy / neuralgia (painful HIV-associated neuropathy) | Repositioning | In development |
| **197** | **Trazodone (15, 155)** | Depression | CNS | Alzheimer’s disease (sleep disorders) | Repositioning | In development |
| **198** | **Valproate (135)** | Epilepsy | CNS | Bipolar disorder | Repositioning | Approved |
| **199** | **Valproate (135)** | Epilepsy | CNS | Epilepsy (microspheres powder adapted for children) | Reformulation | Approved |
| **200** | **Valproate (135)** | Epilepsy | CNS | Migraine disorders | Repositioning | Approved |
| **201** | **Vortioxetine (11, 40, 133)** | Depression (major depressive disorders) | CNS | ADHD | Repositioning | In development |
| **202** | **Zonisamide (10, 86)** | Epilepsy (Partial seizures) | CNS | Parkinson’s disease | Repositioning | Approved |
| **203** | **Zonisamide (10)** | Epilepsy (Partial seizures) | CNS | Dystonia | Repositioning | In development |

Supplementary material references

1. [Aan Het Rot M](http://www.ncbi.nlm.nih.gov/pubmed?term=Aan%20Het%20Rot%20M%5BAuthor%5D&cauthor=true&cauthor_uid=22705040), [Zarate CA Jr](http://www.ncbi.nlm.nih.gov/pubmed?term=Zarate%20CA%20Jr%5BAuthor%5D&cauthor=true&cauthor_uid=22705040), [Charney DS](http://www.ncbi.nlm.nih.gov/pubmed?term=Charney%20DS%5BAuthor%5D&cauthor=true&cauthor_uid=22705040), [Mathew SJ](http://www.ncbi.nlm.nih.gov/pubmed?term=Mathew%20SJ%5BAuthor%5D&cauthor=true&cauthor_uid=22705040). Ketamine for depression: where do we go from here? [Biol Psychiatry.](http://www.ncbi.nlm.nih.gov/pubmed/22705040) 2012 Oct 1;72(7):537-47
2. ALS worldwide website. Link: <http://www.alsworldwide.org/cyclosporin.html>
3. Appleby B, Nacopoulos D, Milano N, Zhong K, Cummings JL. A Review: Treatment of Alzheimer’s disease Discovered in Repurposed Agents. Dement Geriatr Cogn Disord 2013;35:1–22.
4. Ashburn TT, Thor KB. [Drug repositroioning: identifying and developing new uses for existing drugs.](http://www.ncbi.nlm.nih.gov/pubmed/15286734) Nat Rev Drug Discov. 2004 Aug;3(8):673-83
5. Avanir Pharmaceuticals website ([www.avanir.com/nuedexta](http://www.avanir.com/nuedexta))
6. Avigen website (<http://www.avigen.com/av411.php>)
7. Barone P, Poewe W, Albrecht S. Pramipexole for the treatment of depressive symptoms in patients with Parkinson’s disease: a randomised, double-blind, placebo-controlled trial. Lancet Neurol 2010; 9: 573–80.
8. Bastos LF, Coelho MM. Drug Repositioninging: Playing dirty to Kill Pain. Springer International Publishing Switzerland 2013.
9. Black S.E. Toward prevention and treatment for VCID: Past, present, and future. Conference Publication: (var.pagings). 11 (7 SUPPL. 1) (pp P259-P260), 2015.

1. [Bolgár B](http://www.ncbi.nlm.nih.gov/pubmed?term=Bolg%C3%A1r%20B%5BAuthor%5D&cauthor=true&cauthor_uid=24059461), [Arany Á](http://www.ncbi.nlm.nih.gov/pubmed?term=Arany%20%C3%81%5BAuthor%5D&cauthor=true&cauthor_uid=24059461), [Temesi G](http://www.ncbi.nlm.nih.gov/pubmed?term=Temesi%20G%5BAuthor%5D&cauthor=true&cauthor_uid=24059461), [Balogh B](http://www.ncbi.nlm.nih.gov/pubmed?term=Balogh%20B%5BAuthor%5D&cauthor=true&cauthor_uid=24059461), [Antal P](http://www.ncbi.nlm.nih.gov/pubmed?term=Antal%20P%5BAuthor%5D&cauthor=true&cauthor_uid=24059461), [Mátyus P](http://www.ncbi.nlm.nih.gov/pubmed?term=M%C3%A1tyus%20P%5BAuthor%5D&cauthor=true&cauthor_uid=24059461). Drug Repositioninging for treatment of movement disorders: from serendipity to rational discovery strategies. Preliminary technical report.
2. Bortolato B, Miskowiak KW, Köhler CA. Cognitive remission: a novel objective for the treatment of major depression? Bortolato et al. BMC Medicine (2016) 14:9.
3. Brennan JL, Leung JG, Gagliardi JP, Rivelli SK, Muzyk AJ. [Clinical effectiveness of baclofen for the treatment of alcohol dependence: a review.](http://www.ncbi.nlm.nih.gov/pubmed/23869179) Clin Pharmacol. 2013 Jul 3;5:99-107.
4. Bumb JM, Enning F and Leweke FM. Repurposed Drugs for the Treatment of Schizophrenia and Bipolar Disorders. Current Topics in Medicinal Chemistry, 2013, Vol. 13, No. 19.
5. Butchart J, Brook L, Hopkins V, Teeling J, Püntener U, Culliford D, Sharples R, Sharif S, McFarlane B, Raybould R, Thomas R, Passmore P, Perry VH, Holmes C. Etanercept in Alzheimer disease: A randomized, placebo-controlled, double-blind, phase 2 trial. Neurology. 2015 May 26;84(21):2161-8. doi: 10.1212/WNL.0000000000001617. Epub 2015 May 1. Erratum in: Neurology. 2015 Dec 8;85(23):2084.
6. Camargos EF, Louzada LL, Quintas JL, Naves J, Louzada FM, Nobrega OT. Trazodone Improves Sleep Parameters in Alzheimer Disease Patients: A Randomized, Double-Blind, And Placebo-Controlled Study. Am J Geriatr Psychiatry. 2014 Dec; 22(12):1565-74.
7. Chang, Kuo-Hsuan MD, PhD; Wang, Shu-Hui MD, MS. Efficacy and Safety of Topiramate for Essential Tremor A Meta-Analysis of Randomized Controlled Trials. Medicine Volume 94, Number 43, October 2015.
8. ClinicalTrials.gov website. Study of NGX-4010 for the Treatment of Painful HIV-Associated Neuropathy. NCT00321672, Link: <http://clinicaltrials.gov/show/NCT00321672>
9. ClinicalTrials.gov website. A Phase 2b/3 Study to Compare Efficacy and Safety of Masitinib to Placebo in the Treatment of Patients With Primary Progressive or Relapse-free Secondary Progressive Multiple sclerosis. NCT01433497. Phase IIb/III. Link: <https://www.clinicaltrials.gov/ct2/show/NCT01433497?term=nct01433497&rank=1>
10. ClinicalTrials.gov website. A Phase IIa Multi-Center Study of 18F-FDG PET, Safety, and Tolerability of AZD0530 in Mild Alzheimer's Disease. NCT02167256. This Phase II. Link: <https://clinicaltrials.gov/ct2/show/NCT02167256?term=NCT02167256&rank=1>
11. ClinicalTrials.gov website. A Phase III Trial of Nilvadipine to Treat Alzheimer's Disease (NILVAD). NCT02017340 . Phase III. Link: <https://clinicaltrials.gov/ct2/show/NCT02017340?term=nilvadipine&rank=1>
12. ClinicalTrials.gov website. A Phase III, Multi-Center, Double-Blind, Placebo Controlled, Randomized Study of Creatine Monohydrate in Patients With Amyotrophic lateral sclerosis. NCT00069186, Link: <https://clinicaltrials.gov/ct2/show/study/NCT00069186?term=creatine+AND+%28Amyotrophic+Lateral+Sclerosis%29&rank=2>
13. ClinicalTrials.gov website. A Randomised, Double Blind, Placebo Controlled, Single Centre, 60 Week Trial of Exenatide Once Weekly for the Treatment of Moderate Severity Parkinson's Disease. NCT01971242, Link: <https://clinicaltrials.gov/ct2/show/record/NCT01971242?term=Parkinson%27s+Disease+AND+exenatide&rank=1>
14. ClinicalTrials.gov website. A Study of the Effectiveness and Safety of Tetrabenazine MR in Pediatric Subjects With Tourette's Syndrome (TBZ-MR),NCT01133353, Link: <http://clinicaltrials.gov/ct2/show/NCT01133353?term=tetrabenazine&rank=3>.
15. ClinicalTrials.gov website. Albuterol to improve respiratory stranght in SCI. NCT02508311. <https://clinicaltrials.gov/ct2/show/study/NCT02508311>
16. ClinicalTrials.gov website. Buspirone  in the Treatment of 2-6 Year Old Children With Autistic Disorder. NCT00873509. Phase II-III. Link: <https://www.clinicaltrials.gov/ct2/show/study/NCT00873509?term=buspirone&rank=4>
17. ClinicalTrials.gov website. Buspirone Treatment for Marijuana dependence. NCT00875836. Phase IV. Link: <https://www.clinicaltrials.gov/ct2/show/study/NCT00875836?term=buspirone+marijuana&rank=13>
18. ClinicalTrials.gov website. Cilostazol Augmentation Study in Dementia. NCT01409564. Phase IV. Link: <https://www.clinicaltrials.gov/ct2/show/NCT01409564?term=cilostazol&rank=10>
19. ClinicalTrials.gov website. Clinical Trial of Creatine in Amyotrophic lateral sclerosis. NCT00070993, Link: <https://clinicaltrials.gov/ct2/show/study/NCT00070993?term=creatine+AND+%28Amyotrophic+Lateral+Sclerosis%29&rank=3>
20. ClinicalTrials.gov website. Clinical Trial of Creatine in Amyotrophic lateral sclerosis [ALS]. NCT00005674, Link: <https://clinicaltrials.gov/ct2/show/study/NCT00005674?term=creatine+AND+%28Amyotrophic+Lateral+Sclerosis%29&rank=5>
21. ClinicalTrials.gov website. Clinical Trial of Creatine in Amyotrophic lateral sclerosis. NCT00005766, Link: <https://clinicaltrials.gov/ct2/show/record/NCT00005766?term=creatine+AND+%28Amyotrophic+Lateral+Sclerosis%29&rank=6>
22. ClinicalTrials.gov website. Cystagon to Treat Infantile Neuronal Ceroid Lipofuscinosis. NCT00028262. Link: <http://www.clinicaltrials.gov/ct2/show/study/NCT00028262?term=Cysteamine&cond=neuronal+ceroid+lipofuscinoses&rank=1>
23. ClinicalTrials.gov website. Effect of Insulin Sensitizer Metformin on AD Biomarkers. NCT01965756. Phase II. Link: <https://www.clinicaltrials.gov/ct2/show/NCT01965756?term=nct01965756&rank=1>
24. ClinicalTrials.gov website. Efficacy and Safety of Baclofen for Maintenance of Abstinence in Alcohol Dependent Patients (ALPADIR) <https://clinicaltrials.gov/ct2/show/NCT01738282>
25. ClinicalTrials.gov website. Efficacy and Safety Study of Intravenous Progesterone in Patients With Severe Traumatic Brain Injury (SyNAPSe). NCT01143064, Link: <http://clinicaltrials.gov/ct2/show/NCT01143064?term=Progesterone&cond=brain+injury&rank=2&submit_fld_opt=>.
26. ClinicalTrials.gov website. Efficacy Study of NH001 in Vegetative State & Minimally Conscious State Following a Traumatic Brain Injury (NH001-2). NCT00761228,Link:<http://www.clinicaltrials.gov/ct2/show/NCT00761228?term=NH001&cond=vegetative+state+or+minimally+conscious+state+AND+following+a+severe+traumatic+brain+injury&rank=1> (website accessed on 04/11/2014).
27. ClinicalTrials.gov website. Efficacy Study of NH001 in Vegetative State & Minimally Conscious State Following a Traumatic Brain Injury (NH001-2). NCT00761228. Link: <http://www.clinicaltrials.gov/ct2/show/NCT00761228?term=NH001&cond=vegetative+state+or+minimally+conscious+state+AND+following+a+severe+traumatic+brain+injury&rank=1>
28. ClinicalTrials.gov website. Efficiency of Bumetanide in Autistic Children (BUMEA). NCT01078714. Phase III. Link: <https://www.clinicaltrials.gov/ct2/show/NCT01078714?term=NCT01078714&rank=1>
29. ClinicalTrials.gov website. Evaluating Liraglutide in Alzheimer’s Disease. NCT01843075. Phase II. Link: <https://www.clinicaltrials.gov/ct2/show/NCT01843075?term=nct01843075&rank=1>
30. ClinicalTrials.gov website. Evaluation of Mirtazapine and Folic Acid for Schizophrenia: (RECOVERY2). NCT01263080. Phase IV. Link: <https://www.clinicaltrials.gov/ct2/show/NCT01263080?term=NCT01263080&rank=1>
31. ClinicalTrials.gov website. Investigating the Effect of Vortioxetine in Adult ADHD Patients. NCT02327013, Phase II. Link: <https://clinicaltrials.gov/ct2/show/record/NCT02327013?term=vortioxetine+and+attention+deficit&rank=1>
32. ClinicalTrials.gov website. Metformin in Amnestic Mild Cognitive Impairment. NCT00620191. Link: <https://www.clinicaltrials.gov/ct2/show/NCT00620191?term=nct00620191&rank=1>
33. ClinicalTrials.gov website. Mifepristone treatment of alcohol use disorder. NCT02179749. Phase II. Link: <https://clinicaltrials.gov/ct2/show/NCT02179749>
34. ClinicalTrials.gov website. Multicentric Trial of the Treatment of Huntington's Disease by Cysteamine (RP103). NCT02101957, link: <http://www.clinicaltrials.gov/ct2/show/record/NCT02101957?term=Cysteamine&cond=Huntington%27s+disease&rank=1>
35. ClinicalTrials.gov website. N-acetylcysteine Plus Naltrexone for the Treatment of Alcohol Dependence. NCT01214083. Phase II. Link: <https://clinicaltrials.gov/ct2/show/study/NCT01214083>
36. ClinicalTrials.gov website. Ofatumumab Subcutaneous Administration in Subjects With Relapsing-Remitting Multiple sclerosis (MIRROR). NCT01457924. Link: <https://www.clinicaltrials.gov/ct2/show/record/NCT01457924?term=ofatumumab+multiple+sclerosis&rank=2>
37. ClinicalTrials.gov website. Oxcarbazepine for the Treatment of Chronic Peripheral Neuropathic Pain (IMIOXC). NCT01302275. Phase IV. Link: <https://www.clinicaltrials.gov/ct2/show/study/NCT01302275?term=oxcarbazepine&rank=1>
38. ClinicalTrials.gov website. Prazosin for Treatment of Patients With Alcohol Dependence (AD) and Post Traumatic Stress Disorder (PTSD). NCT00744055. Phase III. Link: <https://www.clinicaltrials.gov/ct2/show/study/NCT00744055?term=prazosin+alcohol&rank=4&sect=X870156>
39. ClinicalTrials.gov website. Telmisartan vs. Perindopril in Hypertensive Mild-Moderate Alzheimer's Disease Patients (SARTAN-AD). NCT02085265. Phase II. Link: <https://clinicaltrials.gov/ct2/show/NCT02085265?term=NCT02085265&rank=1>
40. ClinicalTrials.gov website. Use of an Oral Beta-2 Agonist in Persons With Spinal Cord Injury. NCT00755079. Link: <http://www.clinicaltrials.gov/ct2/show/study/NCT00755079?term=albuterol&cond=spinal+cord+injury&rank=1>
41. ClinicalTrials.gov website. Use of Biperiden for the Prevention of Post-traumatic Epilepsy. NCT01048138. Phase III. Link: <https://www.clinicaltrials.gov/ct2/show/NCT01048138?term=biperiden+epilepsy&rank=1>
42. ClinicalTrials.gov website: Antipsychotic Augmentation With L-Dopa. NCT01636037. Phase II. Link: <https://www.clinicaltrials.gov/ct2/show/study/NCT01636037?term=NCT01636037&rank=1>
43. ClinicalTrials.gov website: Caffeine as a Therapy for Parkinson's Disease. NCT01738178. Link: <https://clinicaltrials.gov/ct2/show/NCT01738178?term=NCT01738178&rank=1>
44. ClinicalTrials.gov website: Ceftriaxone in subjects with ALS. NCT00349622. Link: <https://clinicaltrials.gov/ct2/show/results/NCT00349622>
45. ClinicalTrials.gov website: Effect of intranasal insulin on depressive symptoms in Major depressive disorders. NCT00570050. Phase III. Link: <https://clinicaltrials.gov/ct2/show/NCT00570050?term=nct00570050&rank=1>
46. ClinicalTrials.gov website: Safety and Tolerability of Once-daily Oral Aripiprazole in Children and Adolescents With Tourette's Disorder. NCT01727713. Link: <http://www.clinicaltrials.gov/ct2/show/NCT01727713?term=An+Open-Label%2C+Multicenter+Study+Evaluating+the+Safety+and+Tolerability+of+Once-daily+Oral+Aripiprazole+in+Children+and+Adolescents+with+Tourette%E2%80%99s+Disorder&rank=1>
47. Coluzzi F, Mattia C. Chronic non-cancer pain: Focus on once-daily tramadol formulations. Ther Clin Risk Manag. 2007;3(5):819-29.

1. [Corbett A](http://www.ncbi.nlm.nih.gov/pubmed?term=Corbett%20A%5BAuthor%5D&cauthor=true&cauthor_uid=23123941), [Pickett J](http://www.ncbi.nlm.nih.gov/pubmed?term=Pickett%20J%5BAuthor%5D&cauthor=true&cauthor_uid=23123941), [Burns A](http://www.ncbi.nlm.nih.gov/pubmed?term=Burns%20A%5BAuthor%5D&cauthor=true&cauthor_uid=23123941), [Corcoran J](http://www.ncbi.nlm.nih.gov/pubmed?term=Corcoran%20J%5BAuthor%5D&cauthor=true&cauthor_uid=23123941), [Dunnett SB](http://www.ncbi.nlm.nih.gov/pubmed?term=Dunnett%20SB%5BAuthor%5D&cauthor=true&cauthor_uid=23123941), [Edison P](http://www.ncbi.nlm.nih.gov/pubmed?term=Edison%20P%5BAuthor%5D&cauthor=true&cauthor_uid=23123941), [Hagan JJ](http://www.ncbi.nlm.nih.gov/pubmed?term=Hagan%20JJ%5BAuthor%5D&cauthor=true&cauthor_uid=23123941), [Holmes C](http://www.ncbi.nlm.nih.gov/pubmed?term=Holmes%20C%5BAuthor%5D&cauthor=true&cauthor_uid=23123941), [Jones E](http://www.ncbi.nlm.nih.gov/pubmed?term=Jones%20E%5BAuthor%5D&cauthor=true&cauthor_uid=23123941), [Katona C](http://www.ncbi.nlm.nih.gov/pubmed?term=Katona%20C%5BAuthor%5D&cauthor=true&cauthor_uid=23123941), [Kearns I](http://www.ncbi.nlm.nih.gov/pubmed?term=Kearns%20I%5BAuthor%5D&cauthor=true&cauthor_uid=23123941), [Kehoe P](http://www.ncbi.nlm.nih.gov/pubmed?term=Kehoe%20P%5BAuthor%5D&cauthor=true&cauthor_uid=23123941), [Mudher A](http://www.ncbi.nlm.nih.gov/pubmed?term=Mudher%20A%5BAuthor%5D&cauthor=true&cauthor_uid=23123941), [Passmore A](http://www.ncbi.nlm.nih.gov/pubmed?term=Passmore%20A%5BAuthor%5D&cauthor=true&cauthor_uid=23123941), [Shepherd N](http://www.ncbi.nlm.nih.gov/pubmed?term=Shepherd%20N%5BAuthor%5D&cauthor=true&cauthor_uid=23123941), [Walsh F](http://www.ncbi.nlm.nih.gov/pubmed?term=Walsh%20F%5BAuthor%5D&cauthor=true&cauthor_uid=23123941), [Ballard C](http://www.ncbi.nlm.nih.gov/pubmed?term=Ballard%20C%5BAuthor%5D&cauthor=true&cauthor_uid=23123941). Drug Repositioninging for Alzheimer's disease. [Nat Rev Drug Discov.](http://www.ncbi.nlm.nih.gov/pubmed/23123941) 2012 Nov;11(11):833-46.

1. [Corbett A](http://www.ncbi.nlm.nih.gov/pubmed?term=Corbett%20A%5BAuthor%5D&cauthor=true&cauthor_uid=24275851), [Williams G](http://www.ncbi.nlm.nih.gov/pubmed?term=Williams%20G%5BAuthor%5D&cauthor=true&cauthor_uid=24275851), [Ballard C](http://www.ncbi.nlm.nih.gov/pubmed?term=Ballard%20C%5BAuthor%5D&cauthor=true&cauthor_uid=24275851). Drug Repositioninging: an opportunity to develop novel treatments for Alzheimer's disease. [Pharmaceuticals (Basel).](http://www.ncbi.nlm.nih.gov/pubmed/?term=Drug+Repositioning%3A+An+Opportunity+to+Develop+Novel+Treatments+for+Alzheimer%E2%80%99s+Disease) 2013 Oct 11;6(10):1304-21.
2. Curtin F. Multiple sclerosis—new chances. 11th Conference of the European Association for Clinical Pharmacology and Therapeutics, EACPT 2013 Geneva Switzerland. Conference Publication: (var.pagings). 35 (8 SUPPL. 1) (pp e115), 2013.
3. Doctor’s Guide website. Link: <http://www.dgnewsnetwork.com/news/content.nsf/news/852571020057CCF6852571540048442D>.
4. EMA Summary of Product Characteristic. Adasuve. Link: <http://www.ema.europa.eu/docs/en_GB/document_library/EPAR_-_Product_Information/human/002400/WC500139409.pdf>
5. EMA Summary of Product Characteristic. Avastin. (http://www.ema.europa.eu/docs/en_GB/document_library/EPAR_-_Product_Information/human/000582/WC500029271.pdf)
6. EMA Summary of Product characteristic. Buccolam. Link: <http://www.ema.europa.eu/ema/index.jsp?curl=pages/medicines/human/medicines/002267/human_med_001479.jsp&mid=WC0b01ac058001d124>.
7. EMA Summary of Product Characteristic. Exelon. (http://www.ema.europa.eu/docs/en_GB/document_library/EPAR_-_Product_Information/human/000169/WC500032598.pdf)
8. EMA Summary of Product Characteristic. Lemotarda SPC. Link: <http://www.ema.europa.eu/docs/en_GB/document_library/EPAR_-_Product_Information/human/003718/WC500150521.pdf>
9. EMA Summary of Product Characteristic. Lucentis. (<http://www.ema.europa.eu/docs/en_GB/document_library/EPAR_-_Product_Information/human/000715/WC500043546.pdf>)
10. EMA Summary of Product Characteristic. Neudexta ([www.ema.europa.eu/docs/en_GB/document_library/EPAR_-_Product_Information/human/002560/WC500145050.pdf](http://www.ema.europa.eu/docs/en_GB/document_library/EPAR_-_Product_Information/human/002560/WC500145050.pdf))
11. EMA Summary of Product Characteristic. PecFent. Link: <http://www.ema.europa.eu/docs/en_GB/document_library/EPAR_-_Product_Information/human/001164/WC500096493.pdf>
12. EMA Summary of Product characteristic. Zavesca. Link: <http://www.ema.europa.eu/docs/en_GB/document_library/EPAR_-_Product_Information/human/000435/WC500046726.pdf>
13. EMA Summary of Product Characteristic. Zenapax. (http://www.ema.europa.eu/docs/en_GB/document_library/EPAR_-_Product_Information/human/000198/WC500057604.pdf)
14. EMA Summary of Product Characteristics. Tecfidera. <http://www.ema.europa.eu/docs/en_GB/document_library/EPAR_-_Product_Information/human/002601/WC500162069.pdf>
15. EMA. EPAR Summary for the public. MabCampath . Link: <http://www.ema.europa.eu/docs/en_GB/document_library/EPAR_-_Summary_for_the_public/human/000353/WC500025261.pdf>
16. EMA. Public summary of opinion on orphan designation. Progesterone for the treatment of moderate and severe traumatic brain injury. Link: http://www.ema.europa.eu/docs/en_GB/document_library/Orphan_designation/2013/03/WC500139554.pdf.
17. FDA Label. Abilify. (http://www.accessdata.fda.gov/drugsatfda_docs/label/2013/021436s037,021713s029,021729s021,021866s022lbl.pdf)
18. FDA label. Gablofen. <http://www.accessdata.fda.gov/drugsatfda_docs/label/2015/022462s008lbl.pdf>
19. FDA Label. Neurontin. ([www.accessdata.fda.gov/drugsatfda_docs/label/2013/020235s054s055s056,020882s038s039s040,021129s035s036s037lbl.pdf](http://www.accessdata.fda.gov/drugsatfda_docs/label/2013/020235s054s055s056,020882s038s039s040,021129s035s036s037lbl.pdf))
20. FDA label. Plegridy. Link: <http://www.accessdata.fda.gov/drugsatfda_docs/label/2014/125499s000lbl.pdf>
21. FDA label. Risperdal Consta. Link: http://www.accessdata.fda.gov/drugsatfda_docs/label/2010/021346_s31_s35_s38_s39lbl.pdf
22. FDA label. Saphris. Link: <http://www.accessdata.fda.gov/drugsatfda_docs/label/2015/022117s017s018s019lbl.pdf>
23. FDA label. Strattera. Link: <http://www.accessdata.fda.gov/drugsatfda_docs/label/2015/021411s046lbl.pdf>
24. FDA. Lamictal Labeling Changes Overview (http://www.fda.gov/downloads/AdvisoryCommittees/CommitteesMeetingMaterials/PediatricAdvisoryCommittee/UCM234474.pdf)
25. Geldmacher DS, MD; Fritsch T, PhD; McKee J. McClendon, PhD. A Randomized Pilot Clinical Trial of the Safety of Pioglitazone in Treatment of Patients With Alzheimer Disease. Arch Neurol. 2011;68(1):45-50.
26. Google Patente website. Memantine as adjunctive treatment to atypical antipsychotic in schizophrenia patients
    US 20060035888 A1. Link: http://www.google.ch/patents/US20060035888
27. Graul A, Sorbera L, Pina P, Tell M, Cruces E, Rosa E. The Year's New Drugs & Biologics — 2009. Drug News Perspect 23(1), January/February 2010.
28. Green M., Hudson-Farmer K., The Repositioninging revolution: save me from extinction to find me pastures new. Drug Discovery World. Winter 2013. Link: [http://www.ddw-online.com/winter-13-full-articles/p217264-the-Repositioninging-revolution:-save-me-from-extinction-to-find-me-pastures-new.html](http://www.ddw-online.com/winter-13-full-articles/p217264-the-repositioning-revolution:-save-me-from-extinction-to-find-me-pastures-new.html))
29. Grover ND, Limaye RP, Gokhale DV, Patil TR. Zonisamide: a review of the clinical and experimental evidence for its use in Parkinson's disease. Indian J Pharmacol. 2013 Nov-Dec;45(6):547-55.
30. Guo M, Mi J, Qiu-Ming Jiang, Jin-Mei Xu, Matformin may produce antidepressant effects through improvement of cognitive function among depressed patients with Diabetes mellitus. Clinical and Experimental Pharmacology and Physiology (2014) 41, 650–656.
31. Hagerman R, Lauterborn J, Au J, Berry-Kravis E. Fragile X syndrome and targeted treatment trials. [Results Probl Cell Differ.](http://www.ncbi.nlm.nih.gov/pubmed/22009360) 2012;54:297-335.

1. [Hair P](http://www.ncbi.nlm.nih.gov/pubmed?term=Hair%20P%5BAuthor%5D&cauthor=true&cauthor_uid=24000002), [Cameron F](http://www.ncbi.nlm.nih.gov/pubmed?term=Cameron%20F%5BAuthor%5D&cauthor=true&cauthor_uid=24000002), [Garnock-Jones KP](http://www.ncbi.nlm.nih.gov/pubmed?term=Garnock-Jones%20KP%5BAuthor%5D&cauthor=true&cauthor_uid=24000002). Levomilnacipran extended release: first global approval. [Drugs.](http://www.ncbi.nlm.nih.gov/pubmed/?term=hair+2013+AND+milnacipran) 2013 Sep;73(14):1639-45.
2. Hasselmann H.W.W. Ketamine as Antidepressant? Current State and Future Perspectives. Current Neuropharmacology, 2014, 12, 57-70

1. [Heiss WD](http://www.ncbi.nlm.nih.gov/pubmed?term=Heiss%20WD%5BAuthor%5D&cauthor=true&cauthor_uid=22282884), [Brainin M](http://www.ncbi.nlm.nih.gov/pubmed?term=Brainin%20M%5BAuthor%5D&cauthor=true&cauthor_uid=22282884), [Bornstein NM](http://www.ncbi.nlm.nih.gov/pubmed?term=Bornstein%20NM%5BAuthor%5D&cauthor=true&cauthor_uid=22282884), [Tuomilehto J](http://www.ncbi.nlm.nih.gov/pubmed?term=Tuomilehto%20J%5BAuthor%5D&cauthor=true&cauthor_uid=22282884), [Hong Z](http://www.ncbi.nlm.nih.gov/pubmed?term=Hong%20Z%5BAuthor%5D&cauthor=true&cauthor_uid=22282884); [Cerebrolysin Acute Stroke Treatment in Asia (CASTA) Investigators](http://www.ncbi.nlm.nih.gov/pubmed?term=%22Cerebrolysin%20Acute%20Stroke%20Treatment%20in%20Asia%20%28CASTA%29%20Investigators%22%5BCorporate%20Author%5D). Cerebrolysin in patients with acute ischemic stroke in Asia: results of a double-blind, placebo-controlled randomized trial. [Stroke.](http://www.ncbi.nlm.nih.gov/pubmed/?term=Cerebrolysin+in+Patients+With+Acute+Ischemic+Stroke+in+Asia%3A+Results+of+a+Double-Blind%2C) 2012 Mar;43(3):630-6.

1. [Huskamp HA](http://www.ncbi.nlm.nih.gov/pubmed?term=Huskamp%20HA%5BAuthor%5D&cauthor=true&cauthor_uid=19414882), [Busch AB](http://www.ncbi.nlm.nih.gov/pubmed?term=Busch%20AB%5BAuthor%5D&cauthor=true&cauthor_uid=19414882), [Domino ME](http://www.ncbi.nlm.nih.gov/pubmed?term=Domino%20ME%5BAuthor%5D&cauthor=true&cauthor_uid=19414882), [Normand SL](http://www.ncbi.nlm.nih.gov/pubmed?term=Normand%20SL%5BAuthor%5D&cauthor=true&cauthor_uid=19414882). Antidepressant reformulations: who uses them, and what are the benefits? [Health Aff (Millwood).](http://www.ncbi.nlm.nih.gov/pubmed/?term=Antidepressant+Reformulations%3A+Who+Uses+Them%2C+AndWhat+Are+The+Benefits) 2009 May-Jun;28(3):734-45.
2. Ipser JC, Wilson D, Akindipe TO, Sager C, Stein DJ. Pharmacotherapy for anxiety and comorbid alcohol use disorders (Review). Cochrane Library 2015, Issue 1.
3. James D. Berry, Jeremy M. Shefner , Robin Conwit, David Schoenfeld , Myles Keroack. Design and Initial Results of a Multi-Phase Randomized Trial of Ceftriaxone in Amyotrophic lateral sclerosis. PLoS One. 2013; 8(4): e61177.
4. Kim TW. Drug Repositioninging Approaches for the Discovery of New Therapeutics for Alzheimer’s Disease. Neurotherapeutics (2015) 12:132–142.
5. Kleiter I, Gold R. Present and Future therapies in neuromyelitis optica spectrum disorders. Neurotherapeutics (2016) 13:70–83
6. Kour K, Kaur R, Singh J. Memantine for schizophrenia (Protocol). Cochrane Database of Systematic Reviews. Link: <http://onlinelibrary.wiley.com/doi/10.1002/14651858.CD010552/pdf>
7. Levkovitz Y, Alpert C, C.E. Brintz, D. Mischoulon , G.I. Papakostas. Effects of S-adenosylmethionine augmentation of serotonin-reuptake inhibitor antidepressants on cognitive symptoms of Major depressive disorder. European Psychiatry 27 (2012) 518–521.

1. [Li YY](http://www.ncbi.nlm.nih.gov/pubmed?term=Li%20YY%5BAuthor%5D&cauthor=true&cauthor_uid=22494857), [Jones SJ](http://www.ncbi.nlm.nih.gov/pubmed?term=Jones%20SJ%5BAuthor%5D&cauthor=true&cauthor_uid=22494857), Drug Repositioninging for personalized medicine. [Genome Med.](http://www.ncbi.nlm.nih.gov/pubmed/22494857) 2012 Mar 30;4(3):27
2. Lundbeck website. Pipeline. Link: <http://investor.lundbeck.com/pipeline.cfm>
3. Marios C. Papadopoulos, Jeffrey L. Bennett, Alan S. Verkman. Treatment of neuromyelitis optica: state-of-the-art and emerging therapies. Nat Rev Neurol. 2014 September ; 10(9): 493–506.
4. Mason BJ, PhD, Quello S, BA, BS. [Gabapentin treatment for alcohol dependence: a randomized clinical trial.](https://www.ncbi.nlm.nih.gov/pubmed/24190578?dopt=Abstract) JAMA Intern Med. 2014 Jan;174(1):70-7

1. [Mathew SJ](http://www.ncbi.nlm.nih.gov/pubmed?term=Mathew%20SJ%5BAuthor%5D&cauthor=true&cauthor_uid=22303887), [Shah A](http://www.ncbi.nlm.nih.gov/pubmed?term=Shah%20A%5BAuthor%5D&cauthor=true&cauthor_uid=22303887), [Lapidus K](http://www.ncbi.nlm.nih.gov/pubmed?term=Lapidus%20K%5BAuthor%5D&cauthor=true&cauthor_uid=22303887), [Clark C](http://www.ncbi.nlm.nih.gov/pubmed?term=Clark%20C%5BAuthor%5D&cauthor=true&cauthor_uid=22303887), [Jarun N](http://www.ncbi.nlm.nih.gov/pubmed?term=Jarun%20N%5BAuthor%5D&cauthor=true&cauthor_uid=22303887), [Ostermeyer B](http://www.ncbi.nlm.nih.gov/pubmed?term=Ostermeyer%20B%5BAuthor%5D&cauthor=true&cauthor_uid=22303887), [Murrough JW](http://www.ncbi.nlm.nih.gov/pubmed?term=Murrough%20JW%5BAuthor%5D&cauthor=true&cauthor_uid=22303887). Ketamine for treatment-resistant unipolar depression: current evidence. [CNS Drugs.](http://www.ncbi.nlm.nih.gov/pubmed/22303887) 2012 Mar 1;26(3):189-204
2. Medday Pharmaceuticals website. <http://www.medday-pharma.com/research-development/pipeline/md1003/>
3. Medtrack report. Abilify. Link: <https://oneview.medtrack.com/UI/Tools/PE/ProductSummary.aspx?Popup=True&ProductID=903336>
4. Medtrack report. Actinerval. Link: <https://oneview.medtrack.com/UI/Tools/PE/ProductSummary.aspx?Popup=True&ProductID=974175>
5. Medtrack report. AD04. Link: <https://oneview.medtrack.com/UI/Tools/PE/ProductSummary.aspx?Popup=True&ProductID=979567>
6. Medtrack report. Afinitor. Link: <https://oneview.medtrack.com/UI/Tools/PE/ProductSummary.aspx?Popup=True&ProductID=893702>
7. Medtrack report. AGB101. Link: <https://oneview.medtrack.com/UI/Tools/PE/ProductSummary.aspx?Popup=True&ProductID=1054383>
8. Medtrack report. Akrimax Link: <https://oneview.medtrack.com/UI/Tools/PE/ProductSummary.aspx?Popup=True&ProductID=1041546>
9. Medtrack report. Carbamazepin ORTIN. Link: <https://oneview.medtrack.com/UI/Tools/PE/ProductSummary.aspx?Popup=True&ProductID=901777>
10. Medtrack report. Catapres. Link: <https://oneview.medtrack.com/UI/Tools/PE/ProductSummary.aspx?Popup=True&ProductID=898694>
11. Medtrack report. Clodict. Link: <https://oneview.medtrack.com/UI/Tools/PE/ProductSummary.aspx?Popup=True&ProductID=1025747>
12. Medtrack report. Clonicel. Link: <https://oneview.medtrack.com/UI/Tools/PE/ProductSummary.aspx?Popup=True&ProductID=949125>
13. Medtrack report. Clonidine hydrochloride BORSHCHAHIVSKIY. Link: <https://oneview.medtrack.com/UI/Tools/PE/ProductSummary.aspx?Popup=True&ProductID=1061164>
14. Medtrack report. Deplin . Link : <https://oneview.medtrack.com/UI/Tools/PE/ProductSummary.aspx?Popup=True&ProductID=937108>
15. Medtrack report. Insulin. Link: <https://oneview.medtrack.com/UI/Tools/PE/ProductSummary.aspx?Popup=True&ProductID=1070339>
16. Medtrack report. JZP8. Link: <https://oneview.medtrack.com/UI/Tools/PE/ProductSummary.aspx?Popup=True&ProductID=911737>
17. Medtrack report. Ketas. Link: <https://oneview.medtrack.com/UI/Tools/PE/ProductSummary.aspx?Popup=True&ProductID=893534>
18. Medtrack report. Lamictal. Link: <https://oneview.medtrack.com/UI/Tools/PE/ProductSummary.aspx?Popup=True&ProductID=900257>
19. Medtrack report. Lidoderm. Link: https://oneview.medtrack.com/UI/Tools/PE/ProductSummary.aspx?Popup=True&ProductID=898229
20. Medtrack report. LMTX. Link: https://oneview.medtrack.com/UI/Tools/PE/ProductSummary.aspx?Popup=True&ProductID=948139
21. Medtrack report. Masiviera. Link: <https://oneview.medtrack.com/UI/Tools/PE/ProductSummary.aspx?Popup=True&ProductID=895805>
22. Medtrack report. Mexaden. Link: <https://oneview.medtrack.com/UI/Tools/PE/ProductSummary.aspx?Popup=True&ProductID=907514>
23. Medtrack report. Miglustat.
24. Medtrack report. Moclobemide STADA. Link: <https://oneview.medtrack.com/UI/Tools/PE/ProductSummary.aspx?Popup=True&ProductID=978096>
25. Medtrack report. Nuedexta. Link: <https://oneview.medtrack.com/UI/Tools/PE/ProductSummary.aspx?Popup=True&ProductID=894468>
26. Medtrack report. Parkinel. Link: <https://oneview.medtrack.com/UI/Tools/PE/ProductSummary.aspx?Popup=True&ProductID=974590>
27. Medtrack report. Piramed. Link: <https://oneview.medtrack.com/UI/Tools/PE/ProductSummary.aspx?Popup=True&ProductID=907257>
28. Medtrack report. Rexulti. Link: <https://oneview.medtrack.com/UI/Tools/PE/ProductSummary.aspx?Popup=True&ProductID=906131>
29. Medtrack report. Spinalon. Link: <https://oneview.medtrack.com/UI/Tools/PE/ProductSummary.aspx?Popup=True&ProductID=955979>
30. Medtrack report. Tetrabenazine Hetero. Link: <https://oneview.medtrack.com/UI/Tools/PE/ProductSummary.aspx?Popup=True&ProductID=1077326>
31. Medtrack report. Trintellix. Link: https://oneview.medtrack.com/UI/Tools/PE/ProductSummary.aspx?Popup=True&ProductID=896784
32. Medtrack report. USL261. Link: <https://oneview.medtrack.com/UI/Tools/PE/ProductSummary.aspx?Popup=True&ProductID=906161>

1. [Mei H](http://www.ncbi.nlm.nih.gov/pubmed?term=Mei%20H%5BAuthor%5D&cauthor=true&cauthor_uid=22750722), [Xia T](http://www.ncbi.nlm.nih.gov/pubmed?term=Xia%20T%5BAuthor%5D&cauthor=true&cauthor_uid=22750722), [Feng G](http://www.ncbi.nlm.nih.gov/pubmed?term=Feng%20G%5BAuthor%5D&cauthor=true&cauthor_uid=22750722), [Zhu J](http://www.ncbi.nlm.nih.gov/pubmed?term=Zhu%20J%5BAuthor%5D&cauthor=true&cauthor_uid=22750722), [Lin SM](http://www.ncbi.nlm.nih.gov/pubmed?term=Lin%20SM%5BAuthor%5D&cauthor=true&cauthor_uid=22750722), [Qiu Y](http://www.ncbi.nlm.nih.gov/pubmed?term=Qiu%20Y%5BAuthor%5D&cauthor=true&cauthor_uid=22750722). Opportunities in systems biology to discover mechanisms and repurpose drugs for CNS diseases. [Drug Discov Today.](http://www.ncbi.nlm.nih.gov/pubmed/?term=Opportunities+in+systems+biology+to+discover+mechanisms+and+repurpose+drugs+for+CNS+diseases) 2012 Nov;17(21-22):1208-16
2. MHRA Summary of Product Characteristic. Haldol Decanoate. <http://www.mhra.gov.uk/home/groups/spcpil/documents/spcpil/con1472189995453.pdf>
3. MHRA Summary of Product characteristic. Midazolam 1mg/ml, solution for injection. <http://www.mhra.gov.uk/home/groups/spcpil/documents/spcpil/con1469161367684.pdf>
4. MSI Methylation Sciences (developer of S-Adenosyl methionine) website ([www.methylationsciences.com](http://www.methylationsciences.com))
5. Mucke HAM, Drug Repositioninging: Extracting Added Value from Prior R&D Investments. InsightPharmaReports .com. Published in July 2010 by Cambridge Healthtech Institute.
6. Multiple Sclerosis Society website. Daclizumab (http://www.mssociety.org.uk/ms-research/new-and-potential-treatments/daclizumab)
7. Murteira S, Millier A, Ghezaiel Z, Lamure M, Drug reformulations and Repositioninging in the pharmaceutical industry and their impact on market access: regulatory implications. Journal of Market Access & Health Policy 2014, 2: 22813 - <http://dx.doi.org/10.3402/jmahp.v2.22813>
8. *Murteira S., Ghezaiel Z., Karray S., Lamure M.* Drug reformulations and Repositioninging in pharmaceutical industry and its impact on market access: reassessment of nomenclature. Journal of Market Access & Health Policy 2013, **1**: 21131 - <http://dx.doi.org/10.3402/JMAHP.v1i0.21131>
9. Novac N., [Challenges and opportunities of drug Repositioninging.](http://www.ncbi.nlm.nih.gov/pubmed/23582281) Trends Pharmacol Sci. 2013 May;34(5):267-72.
10. Nygaard HB, Wagner AF, Bowen GB. A phase Ib multiple ascending dose study of the safety, tolerability, and central nervous system availability of AZD0530 (saracatinib) in Alzheimer’s disease. Nygaard et al. Alzheimer's Research & Therapy (2015) 7:35.
11. OCD-UK website – fluvoxamine ([www.ocduk.org/fluvoxamine](http://www.ocduk.org/fluvoxamine))
12. OCD-UK website – paroxetine (http://www.ocduk.org/paroxetine)
13. One Blockbuster Drug Explains A Lot About Our Out-Of-Control Healthcare Costs. <http://www.businessinsider.com.au/price-difference-lucentis-and-avastin-2014-6>
14. Padhy BM, Gupta YK., Drug Repositioninging: Re-investigating existing drugs for new therapeutic indications. [J Postgrad Med.](http://www.ncbi.nlm.nih.gov/pubmed/?term=padhy+AND+repositioning) 2011 Apr-Jun;57(2):153-60
15. Pich EM, Collo G. Pharmacological targeting of dopamine D3 receptors: Possible clinical applications of selective drugs. European Neuropsychopharmacology(2015) 25, 1437–1447.
16. Potter DE and Choudhury M. Ketamine: repurposing and redefining a multifaceted drug. Drug Discovery Today, Volume 19, Number 12, December 2014.
17. Prescribing information. Fumaderm . Link: <https://www.wuensche.synology.me/Wordpress/wp-content/uploads/Fumarderm.pdf>
18. Rong Xu, QuanQiu Wang. PhenoPredict: A disease phenome-wide drug Repositioninging approach towards schizophrenia drug discovery. Journal of Biomedical Informatics 56 (2015) 348–355.
19. Rumore MM, PharmD, JD, MS, LLM. Medication Repurposing in Pediatric Patients: Teaching Old Drugs New Tricks. J Pediatr Pharmacol Ther 2016 Vol. 21 No. 1.
20. Scahill L, Leckman JF, Schultz RT, Katsovich L, Peterson BS. A placebo-controlled trial of risperidone in Tourette syndrome. Neurology. 2003; 69:1130–5
21. Scoralick FM, Camargos EF, Freitas MP, Nóbrega OT. Outpatient treatment of sleep disorders in Alzheimer patients. Einstein (Sao Paulo). 2015 Jul-Sep;13(3):430-4.
22. Sekhon BS., Repositioninging drugs and biologics: Retargeting old/existing drugs for potential new therapeutic applications. Journal of Pharmaceutical Education and Resaerch 06/2013; 4(1):1-15.
23. Shirani A, Okuda DT, Stüve O. Therapeutic Advances and Future Prospects in Progressive Forms of Multiple sclerosis. Neurotherapeutics (2016) 13:58–69.
24. Shirani A, Okuda DT, Stuve O. Research trends in therapeutics for progressive forms of Multiple sclerosis: a review of trials in progress and future prospects. Conference Publication: (var.pagings). 23 (11 SUPPL. 1) (pp 770-771), 2015.
25. *Smith RB.*, Repositioninged drugs: integrating intellectual property and regulatory strategies. Drug Discovery Today: Therapeutic Strategies. 2011; Vol. 8, No. 3–4: 131-137
26. Steinman L. Development of therapies for autoimmune disease at Stanford: a tale of multiple shots and one goal. Immunol Res (2014) 58:307–314.
27. Terevnikov V. Clinical effects of mirtazapine added to first generation antipsychotics in schizophrenia. Academic Dissertation 2013. Link: https://helda.helsinki.fi/bitstream/handle/10138/38492/clinical.pdf?sequence=1

1. [Tobinick E](http://www.ncbi.nlm.nih.gov/pubmed?term=Tobinick%20E%5BAuthor%5D&cauthor=true&cauthor_uid=20518613), Perispinal etanercept: a new therapeutic paradigm in neurology. [Expert Rev Neurother.](http://www.ncbi.nlm.nih.gov/pubmed/?term=Perispinal+etanercept%3A+a+new+therapeutic+paradigm+in+neurology) 2010 Jun;10(6):985-1002.
2. U.S. Department of Health and Human Services. National Institutes of Health. National Institute of Mental Health. Anxiety Disorders. NIH Publication no. 093879. 2009 (<http://www.nimh.nih.gov/health/publications/anxiety-disorders/nimhanxiety.pdf>)
3. Vesterinen HM, Connick P, Irvine CMJ, Sena ES. Drug Repurposing: A Systematic Approach to Evaluate Candidate Oral Neuroprotective Interventions for Secondary Progressive Multiple sclerosis. PLOS ONE | DOI:10.1371/journal.pone.0117705 April 9, 2015.
4. Villapol S and Saavedra JM. Neuroprotective Effects of Angiotensin Receptor Blockers. American Journal of Hypertension 28(3) March 2015.

1. [Wheless JW](http://www.ncbi.nlm.nih.gov/pubmed?term=Wheless%20JW%5BAuthor%5D&cauthor=true&cauthor_uid=23112237), [Phelps SJ](http://www.ncbi.nlm.nih.gov/pubmed?term=Phelps%20SJ%5BAuthor%5D&cauthor=true&cauthor_uid=23112237). Clobazam: a newly approved but well-established drug for the treatment of intractable epilepsy syndromes. [J Child Neurol.](http://www.ncbi.nlm.nih.gov/pubmed/?term=Wheless+2013+AND+clobazam) 2013 Feb;28(2):219-29.
2. Winhusena T, Bradyb KT, Stitzer M. Evaluation of buspirone for relapse-prevention in adults with cocaine dependence: An efficacy trial conducted in the real world. Contemp Clin Trials. 2012 September ; 33(5): 993–1002.
3. Yang CS, Zhang LL. Topiramate for Tourette’s Syndrome in Children: A Meta-Analysis. Pediatric Neurology 49 (2013) 344e350
4. Zaccara G, Schmidt D. Do traditional anti-seizure drugs have a future? A review of potential anti-seizure drugs in clinical development. Pharmacological Research 104 (2016) 38–48.
5. Zarate CA, Manji HK. [Protein Kinase C Inhibitors: Rationale for Use and Potential in the Treatment of Bipolar Disorder](http://www.ncbi.nlm.nih.gov/pmc/articles/PMC2802274/). CNS Drugs. 2009; 23(7): 569–582.
6. Zarate CA, Manji HK. [Putative Drugs and Targets for Bipolar Disorder](http://www.ncbi.nlm.nih.gov/pmc/articles/PMC2754305/). [Mt Sinai J Med. 2008 May–Jun; 75(3): 226–247](http://www.ncbi.nlm.nih.gov/entrez/eutils/elink.fcgi?dbfrom=pubmed&retmode=ref&cmd=prlinks&id=18704977)
7. Zogenix website. Pipeline. Link: <http://www.zogenix.com/c/pipeline/index.php>
